# Using the Design Thinking Process to Co-create a New, Interdisciplinary Design Thinking Course to Train 21st Century Graduate Students

**DOI:** 10.3389/fpubh.2021.777869

**Published:** 2022-01-11

**Authors:** Emily Rose Skywark, Elizabeth Chen, Vichitra Jagannathan

**Affiliations:** Department of Health Behavior, Gillings School of Global Public Health, University of North Carolina, Chapel Hill, NC, United States

**Keywords:** co-creation, course development, social innovation, curricular development, complex problem solving

## Abstract

**Background:** Our instructional team at the The University of North Carolina at Chapel Hill led an innovative project that used IDEO.org's design thinking process to create a brand-new interdisciplinary graduate course, housed in the school of public health, titled *Design Thinking for the Public Good*. We offer our course design process as a case study of the use of design thinking for course design.

**Methods:** We collected data and generated insights through a variety of inspiration, ideation, and implementation design thinking methods alongside members of our three stakeholder groups: (1) faculty who teach or have taught courses related to design thinking at our higher education institution; (2) design thinking experts at ours and other institutions and outside of higher education; and (3) graduate students at our institution.

**Results:** We learned that interdisciplinary design thinking courses should include growth-oriented reflection, explicit group work skills, and content with a real-world application.

**Conclusions:** Our course design process and findings can be replicated to design courses regardless of area of study, level, or format.

## Introduction: Background and Rationale for the Educational Activity Innovation

The purpose of this case study is to describe our design thinking approach for course development and provide sufficient detail so that other educators can learn from our experience and replicate or adapt this process for their own course development projects. Design thinking is a repeatable, creative approach to problem solving that synthesizes what is desirable to real stakeholders, technologically feasible, and economically sustainable (1, 2). By beginning with the needs of stakeholders and engaging in short iteration cycles, design thinking allows for solutions that roll out smoothly with lasting impact (1, 3). This is an especially important tool in public health because the human-centered real-world solutions that are generated from the design thinking process have the potential to be systemic solutions to the wicked social problems our students want to solve (4). A systematic review of 24 articles designed with the purpose of determining how applying design thinking to health care could enhance innovation, efficiency, and effectiveness by increasing focus and patient and provider needs found that design thinking has promising implications for the acceptability and effectiveness of health care intervention development, implementation, and dissemination because patients and providers are actively engaged in an iterative design process (5).

This project's goal was to create a new *Design Thinking for the Public Good* course for graduate students at the The University of North Carolina at Chapel Hill (UNC-CH) which would serve as the core course for a new, interdisciplinary *Innovation for the Public Good* graduate certificate. The certificate is sponsored jointly by the schools of public health, public policy, and education. UNC-CH and other innovative universities often do not have a coordinated interdisciplinary course offering that is broadly accessible to students outside of the business and STEM programs nor do they offer pan-campus programs in social innovation. Especially for public health students, coordinated programs like this are becoming more necessary to shift from models that are predominantly knowledge-acquisition-based to models that include “broader experiences involving entrepreneurial thinking that maximizes career opportunities for graduates in a world where the scale of change is increasingly more complex, unpredictable and uncertain” (6). The new, interdisciplinary graduate certificate at UNC-CH will offer a program that focuses on teaching these methods and tools which are necessary for innovation for the public good and solving complex social problems.

## Pedagogical Frameworks, Pedagogical Principles, Competencies/Standards Underlying the Educational Activity

The instructional team used the design thinking process for course development. Other common course design methods include backward design, integrated course design, Design Based Implementation Research (DBIR), social constructivist philosophy and Universal for Design Learning (UDL) (7–11). Design thinking for course design is different from these other approaches because it begins with specific parameters (e.g., design thinking course for graduate social innovators) rather than specific learning objectives, as backward design and integrated course design do. The undefined objectives allow the course to be driven by the needs of stakeholders and for the design to happen alongside the blooming understanding of these needs. This grounding in diverse stakeholders is like the process used in DBIR, but DBIR is intended to improve teaching and learning at scale (11). Moreover, the collaborative learning environment is like the social constructivist philosophy, which involves the collaborative discovering of trusts, however the design thinking model leads to a product that can be generalized for all students which is necessary when course content cannot be customized (9). Design thinking enhances the UDL framework because designers can deeply empathize with students to develop specific strategies for representation, action and expression, and engagement (10). The details of our design thinking course development process and lessons learned may be especially helpful to those who have not engaged with an explicit course development process before.

Creating a course in this way, and publicly disseminating the methods and lessons learned, is a novel addition to literature focused on innovation in higher education. A review of 31 available design thinking and design thinking pedagogy articles offered insight into the potential of design training outside of traditional design, business and STEM, as well as the obstacles one might face in both teaching and learning design thinking (4, 12–40). We found no examples of using the design thinking process to co-create an interdisciplinary design thinking course with stakeholders. Further, we reviewed the websites of the top 50 United States colleges and universities on Reuter's list of The World's Most Innovative Universities (41) to see if a course like ours existed at other innovative universities. This search revealed that most design thinking courses and programs are housed in design, STEM, or business schools. While we found some programs that were not umbrellaed within design, STEM, or business schools, we found no courses like ours that were interdisciplinary, graduate-level design thinking courses housed in a public health school. Upon a more extensive review of universities with options specifically for design thinking and healthcare, we found only eight other universities in the US with dedicated courses, programs or centers.

To utilize consistent methods our instructional team decided to apply IDEO.org's design thinking process, mindsets, and methods available in *The Field Guide for Human-Centered Design* (2). IDEO.org calls their version of design thinking “human-centered design” and defines it as a “creative approach to problem solving…that starts with the people you're designing for and ends with new solutions that are tailor made to suit their needs” (2). IDEO.org's human-centered design process moves through convergent and divergent cycles in three phases: inspiration, ideation, and implementation (2). The inspiration phase provides an opportunity for problem definition and building empathy through various activities that allow the design team to experience the problem for which a solution is needed and deeply engage with stakeholders who are true experts. The goal of this phase is not to develop a solution but to deeply understand the experience of stakeholders with the problem, including pain points or workarounds they have embraced (1, 2). In the ideation phase, the design team uses the insights from empathy work with stakeholders to generate many ideas for how to solve the problem. In the implementation phase, ideas are tested through quick prototypes, and feedback is generated to inspire iteration (1, 2). This case study will cover our methods, results, and lessons learned from the inspiration and ideation phases of our course development project.

## Learning Environment (Setting, Students, Faculty); Learning Objectives; Pedagogical Format

Our design team consisted of three team members with diverse backgrounds and all of whom would have a role on the teaching team for the new course in Spring 2021. Dr. Elizabeth Chen is a faculty member in the School of Public Health at UNC-CH, was formally trained in design thinking by IDEO, and has a design thinking leadership role at the University. Ms. Vichitra Jagannathan is a co-founder of a social innovation lab that uses design thinking to design public health solutions with rural communities in a southern state. Ms. Jagannathan was trained in design thinking by IDEO as well, holds engineering and business degrees, and was a former high school teacher along with Dr. Chen. The IDEO training that Dr. Chen and Ms. Jagannathan received guided this design process. Ms. Emily Rose Skywark was a Master of Public Health Student at the School of Public Health at UNC-CH with undergraduate degrees in history and international relations, consulting experience, and an interest in creative problem-solving in public health.

The course was designed for 50 graduate student, some of which would come from the CIPG program and others who would join the class from schools across the university. Aware that students would come from diverse programs with varying requirements, the course was scheduled for a 3 h block, once a week, from 5:30 pm-8:30 pm, after standard close-of-business. Due to the timing of the Covid-19 pandemic, we became aware that the course we were designing would need to be adaptable for delivery either virtually or in person. Eventually, it would be delivered entirely over Zoom supported by a course website.

The overall goal of this course was to facilitate student application of the mindsets, methods, and process associated with design thinking (i.e., human-centered design) to solve real world problems. Specifically, the learning objectives were that by the end of the course, students will be able to independently:

1. Identify how to center innovation designs as a response to the voice, experiences, wishes, and aspirations of those most directly impacted by innovation
2. Develop an understanding of one's own experiences, intentions, strengths and limitations, motivations, and biases as a changemaker relative to the impacted audiences
3. Identify, define, and clearly analyze a problem, recognize opportunities, challenges, and the assets of communities as they address the problem, and generate optimal solutions through the application of social innovation in practice
4. Understand how the context in which a problem is located, and solution is imagined shapes and impacts the innovation design and implementation process
5. Understand how to effectively engage stakeholders in co-design, implementation, evaluation, and adaptive learning associated with the innovation.

## Results to Date/Assessment (Processes and Tools; Data Planned or Already Gathered)

Our team began first with inspiration. This stage took us 3 months to complete, with some overlap between inspiration and ideation throughout the third month. Due to the emergence of the coronavirus pandemic during this stage, we adjusted our vision for the course to ensure that it could be delivered successfully both in-person and virtually, allowing for additional depth in our recommendations which include insights relevant to both forms. We also transitioned from in-person activities to virtual meetings, proving that this process can succeed in either format. We then moved to the ideation phase, during which we began to make sense of our insights, generated ideas, and developed solutions we could test. The inspiration stage took us about two and a half months, with 1 month overlapping with the end of the inspiration stage. Overall, the inspiration and ideation process took us about four and a half months which spanned the length of a semester. While we had opportunities to begin to implement through testing and iteration in the ideation stage, our true implementation phase was the first iteration of this course taught in Spring 2021. We present our detailed methods and lessons learned so that others may use a similar design thinking process to co-create with fidelity innovative courses that center stakeholders' experiences and needs. As we describe our course development process, we underline each of the IDEO.org human-centered design methods. A brief overview of our timeline and the IDEO.org methods we used is presented in Table 1. For further information about the method descriptions and the findings for each specific activity (see Appendix A).

**Table 1:** Course development process overview.

**Inspiration**	**Ideation**
*3 months*	*1.5 months*
Frame your design challenge	Download your learnings
Build a team	Share inspiring stories
Recruiting tools	Find themes
Secondary research	HMW
Expert interviews	Brainstorm with brainstorming rules
Extremes and mainstreams	Bundle ideas
Immersion	Design principles
Analogous inspiration	Rapid prototyping
	Create a concept
	Create insight statements
	Co-creation session
	Determine what to prototype
	Rapid prototyping
	Get feedback

### Inspiration

To begin our course design process, we formed the team discussed in the previous section Each team member independently completed a Frame Your Design Challenge worksheet, which allowed for the team to come together to understand the design challenge and collectively revise it. This activity generated our guiding How Might We (HMW) question: “HMW recruit and equip an interdisciplinary team of UNC-CH grad students to apply design thinking approaches, tools, and mindsets to solve diverse and complex problems and share their process and insights with community members?”

We then conducted *second*ary research to inform our other inspiration activities. We gathered and reviewed 24 design thinking and innovation syllabi from UNC-CH faculty who taught courses like ours and reviewed 13 published, peer-reviewed articles about design thinking training, research, and pedagogy. These two waves of secondary research helped us generate new questions to consider related to the student experience, design thinking's application to multiple disciplines, the course structure, and course content. See Appendix A for the specific insights this secondary research generated.

We also sought an analogous inspiration experience that was non-linear and would inspire members of our instructional team to accept ambiguity as design thinking is a non-linear (e.g., you can advance to the ideation phase and then go back to the inspiration phase) and sometimes ambiguous process. To experience these emotions in a setting outside of the classroom we chose to participate in an in-person escape room where the three of us had to solve a series of puzzles and riddles to “escape” a themed room together in 1 h or less. Our team entered with guiding questions and paid attention to how we felt throughout the experience. This generated insights about team structure, the potential to “level-up” with a follow-on course that goes into further depth, the classroom environment, and ensuring that the same information is provided to all teams. See Appendix A for the specific insights this analogous inspiration generated.

#### Interviews

To learn from the experiences of design thinking experts and gather graduate students' wants and needs for this new course, we conducted interviews with five design thinking faculty at UNC-CH, three design thinking program leads at other higher education institutions, two expert design thinking practitioners from the community, and nine UNC-CH graduate students.

##### Interviews With UNC-CH Faculty who Teach Design Thinking

A list of 24 UNC-CH faculty who teach design thinking was collected. Five interdisciplinary faculty members were selected for interviews after they responded to an initial survey, we sent about teaching design thinking (see Appendix A for additional methods). In the interviews UNC-CH faculty stressed the importance of teaching design thinking mindsets to students even more so than teaching explicit methods. The design thinking mindsets would allow students to empathize, solve complex problems, be creative, etc. throughout their lives. In addition, the interviews revealed that faculty believed that sufficient time for reflection aids in the development and measurement of these mindsets. They generally agreed that inspiration/empathy were the hardest yet most important phases of the design thinking process. In addition to interviews with UNC-CH faculty, two members of the instructional team engaged in immersion activities and observed faculty teaching two design thinking courses at UNC-CH. Results from our immersion activities are further detailed in Appendix A.

##### Interviews With Design Thinking Leads at Other Higher Education Institution

A list of eight design thinking program leads at the top 50 Most Innovative Universities in the United States was generated (41). Three design thinking program leads consented to participate in semi-structured interviews. The interviews helped us understand what has worked well in design thinking education, what challenges persist, how to best measure outcomes, and what to expect of students. The interviews affirmed that teaching design thinking is hard, and the classroom environment matters. It was clear that students need a gradual release of new information, as design thinking is new and unfamiliar at first, as well as a scaffolding of skills (42). Across courses, they echoed UNC-CH faculty in emphasizing that the empathy stage of the design thinking process is both the most labor intensive and the most important. Additional insights are reflected in Appendix A.

##### Interviews With Expert Design Thinking Practitioners in the Community

Two community members who the instructional team knew because of their use of design thinking were interviewed. Five other community members took part in a group interview panel organized by Dr. Chen and facilitated by Ms. Skywark. Graduate students had the opportunity to ask questions during this panel as well. These community members provided insight into the potential applications of design thinking for students, and the opportunities/challenges that exist around community engagement. Additional insights are reflected in Appendix A.

##### Interviews With UNC-CH Graduate Students

In addition to speaking with design thinking experts, we decided to speak to graduate students who showed interest in taking the course. Recruitment materials were created with this specific group as the target. Of the 43 students who showed interest in our course by completing an online survey, nine graduate students were interviewed. To choose which graduate students to interview we examined extremes and mainstreams to ensure we selected students with a diversity of degree programs, design thinking experience, and demographics. Students with no design thinking experience were selected as the mainstreams (typical students) and students with a lot of design thinking experience were selected as the extremes (atypical students). From these interviews we learned that graduate students were attracted to design thinking because of its non-traditional problem-solving approach. Though design thinking is new and unfamiliar at first, students seemed to find an anchoring point from their own experiences or training through which they connect to design thinking. This was evident in connections made to similar creative processes and in the frequent reference to the Henry Ford quotation: “If I had asked people what they wanted, they would have said faster horses.”

#### Ideation

**Figure 1:**
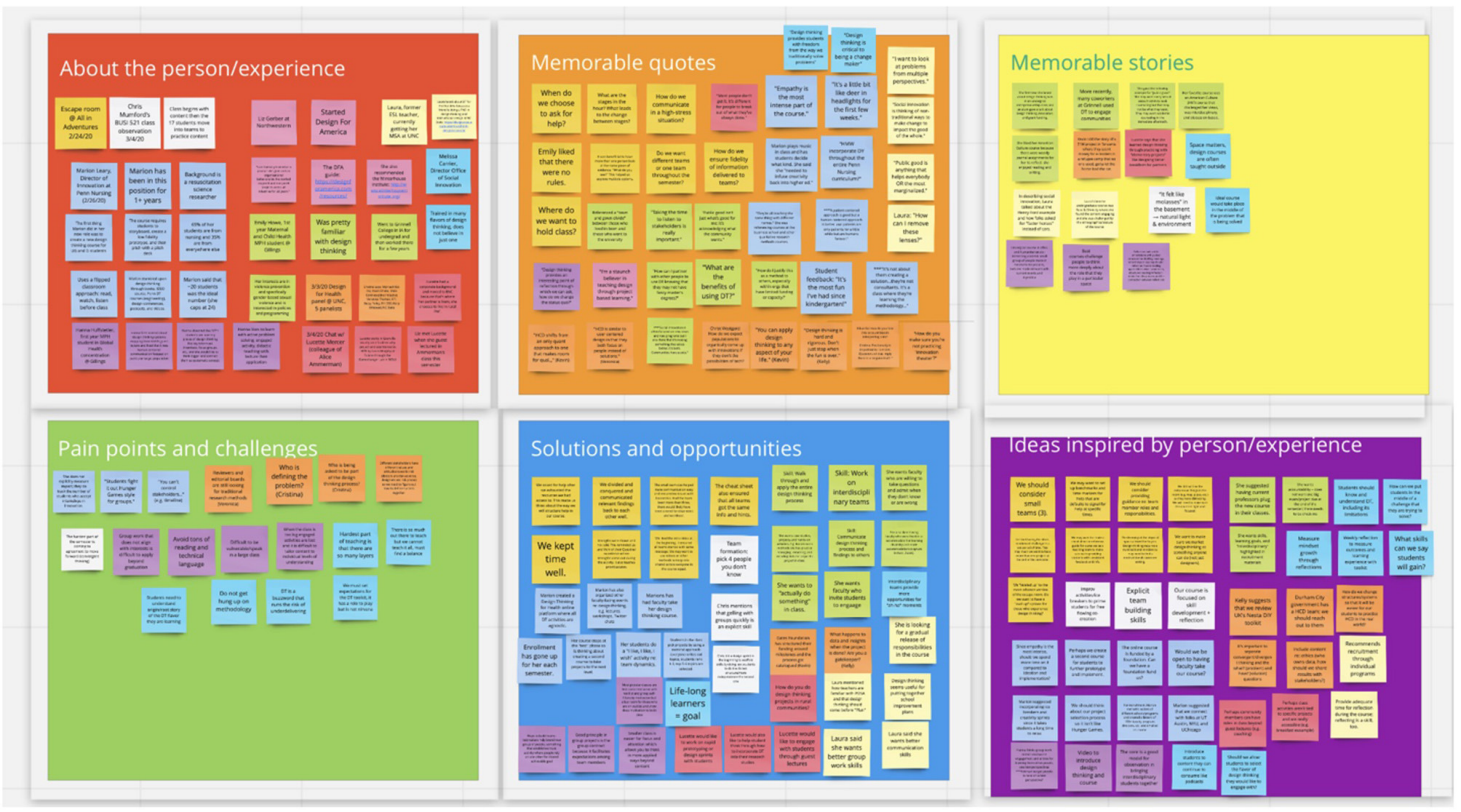
Example Miro board with downloaded learning.

To turn the insights gained into tangible ideas, we moved through a series of activities in the ideation phase of the design thinking process. As we transitioned there were moments when the inspiration and ideation phases occurred simultaneously, as insights generated new questions that were explored through further inspiration methods. The first ideation method we used was download learnings. We created three sets of boards in Miro with hundreds of virtual sticky notes from our interviews, analogous experiences, immersion, and secondary research. Sticky notes were organized into six categories per board: about person/experience, memorable quotes, memorable stories, pain points, solutions/opportunities, ideas generated by person/experience. See Figure 1 for an example Miro board. We met weekly to review new additions to the boards based on inspiration activities and to share inspiring stories. This process was non-linear, and we downloaded and reflected in three waves throughout the ideation process and sometimes went back to complete additional inspiration activities.

**Figure 2:**
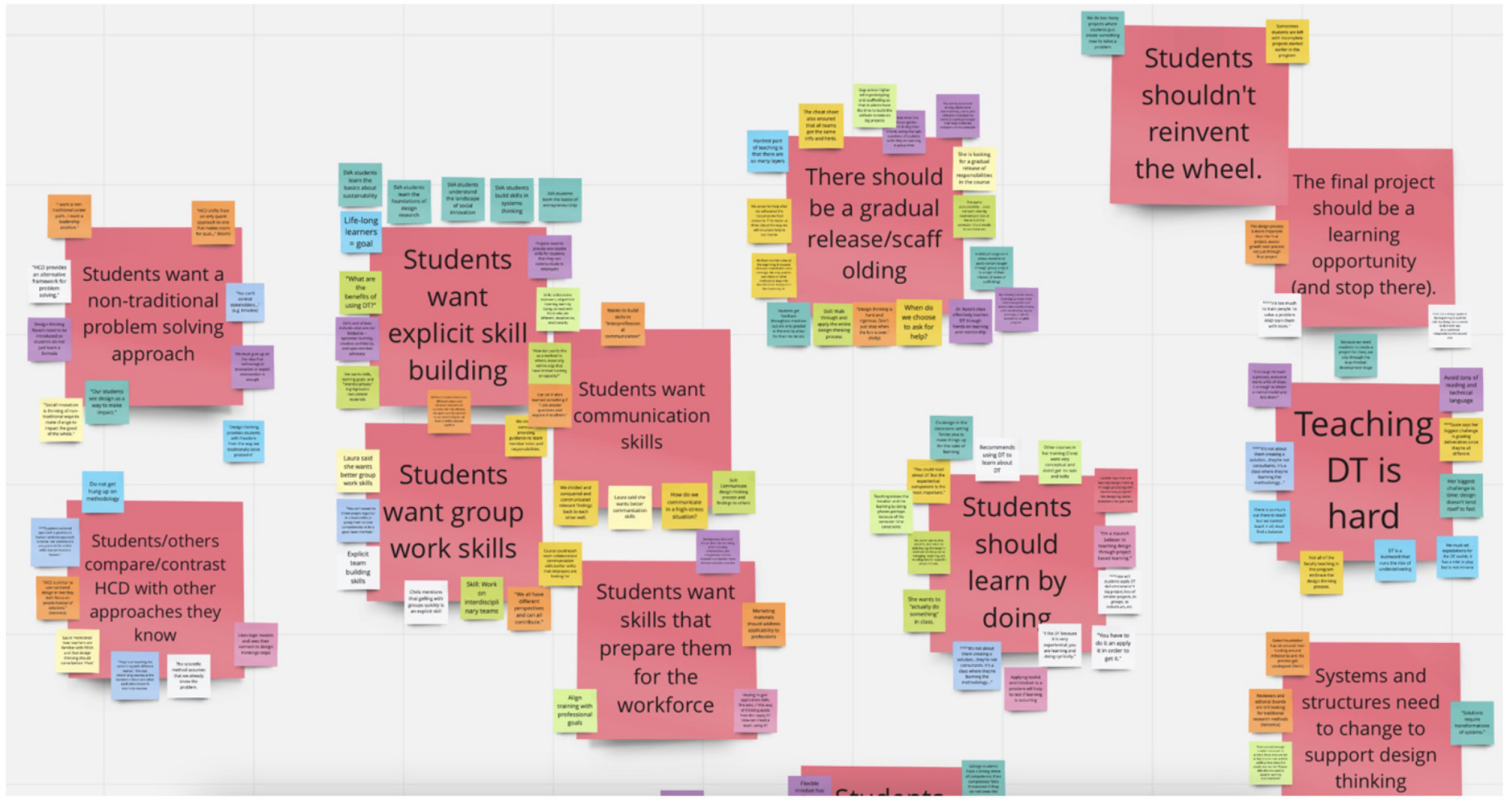
Example Miro board with clusters and themes.

This iteration strengthened the insights generated from learnings and our team's ability to find themes. After all learnings were downloaded, we made a second copy of each Miro board from which sticky notes could be grouped into themes through bundling. See Figure 2 for an example Miro board where clusters of sticky notes were grouped together and assigned a theme. We saw three sets of key themes emerge: (1) students wanted explicit skill-building; (3) students wanted a non-traditional problem-solving approach; and (4) the design thinking course needed to be intentionally designed and delivered, with skills scaffolded for students throughout. For more information about these three sets of key themes (see Appendix A).

After identifying key themes, we synthesized our findings by creating insight statements that could point the way forward. Insight statements transform themes into short statements that feel like core research insights (2). We wrote four insight statements that informed new HMW (HMW) questions and design principles. Our key insights, which became our design principles for this course, are that (1) design thinking should be taught as a tool in a problem-solving toolkit, (3) we must ensure that graduate students, community partners and the teaching team learn and mutually benefit, (4) group work is an art and a science; and that (5) individual reflection should be part of the design process for mindset development. Using these insights and the HMW questions generated during individual brainstorming, we developed five new HMW questions:

1. HMW situate design thinking among other problem-solving approaches throughout the course?
2. HMW provide students with real-life learning experiences, while ensuring that community partners' expectations are met?
3. HMW effectively support and equip students to engage in interdisciplinary collaboration within and outside traditional university environments?
4. HMW ensure that students both experience a feeling of mastery of the design process (scaffolded structure that has opportunities for leveling up, growth mindset) to a depth that they can apply in the future, while also creating space for adequate reflection (on lenses, biases, ethics, power, positionality)?
5. HMW create a learning environment that is both physically and emotionally inviting for all types of thinkers?

Inspired by the tensions identified in these questions, our team thought it was necessary to brainstorm solutions to each of the five new HMW questions. A new Miro board was created, with a column for each question. Through independent and team brainstorms using brainstorming rules, 132 potential solutions were generated. After the individual brainstorm and team debrief, similar ideas were bundled using Miro. Similar solutions that all team members had generated independently were bundled together into “things that will be true for our course,” including that we will create opportunities for students to give/receive feedback on group work, and students should reflect on growth mindset, failure, and equity.

**Figure 3:**
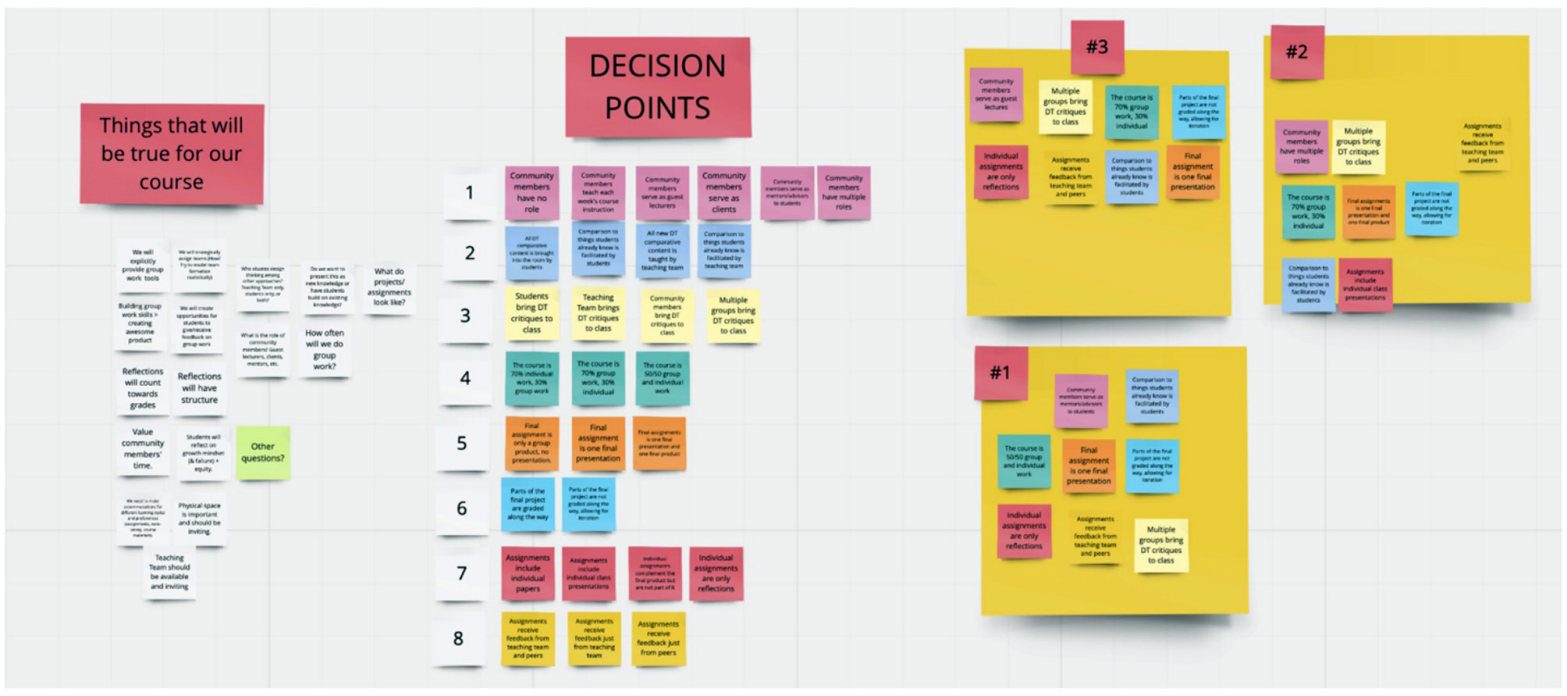
Decision points on Miro board.

Coordinated insights about solutions were polished into one to two design principles for each HMW statement (see Appendix A). These design principles served as our guardrails and held us accountable as we developed the new course. Where there were different solutions and no clear consensus, decision points emerged, and we created eight decision point categories as listed in Appendix A. It was in working through these decision points that we were able to create a concept for the course. For each decision point, possible options were brainstormed. These options were laid out on the periphery of the Miro board in eight horizontal rows corresponding with each decision point, presented in Figure 3.

**Figure 4:**
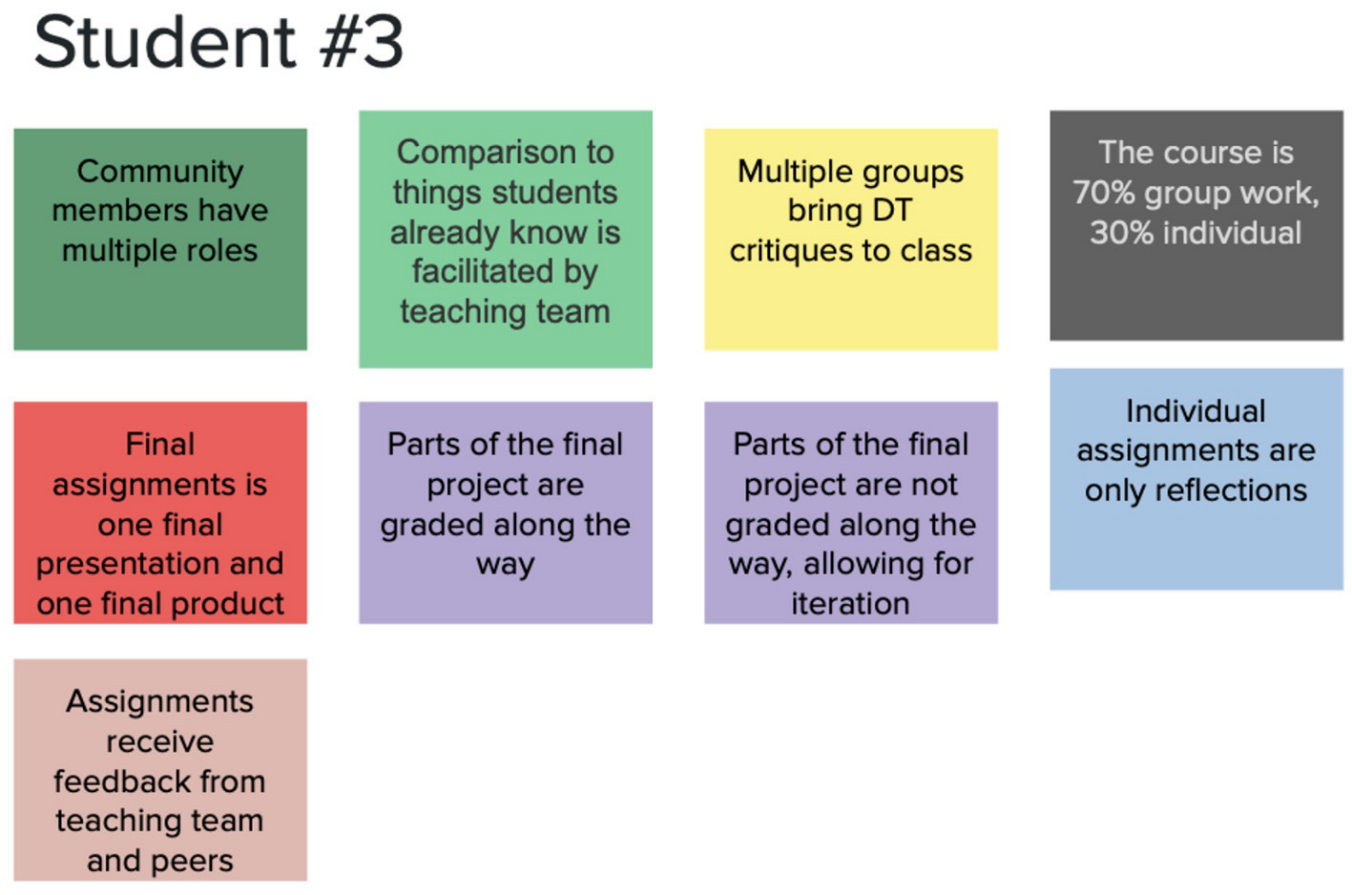
Graduate student course concept from co-creation session.

To create a concept for the course, a large virtual sticky note was created for each team member. Team members independently created a concept by selecting one option from each decision point and moving it onto their sticky note. After all three course concepts were created, the instructional team then compared their concepts, looking for points of agreement. To settle points of disagreement, we saw an opportunity to stay grounded with our stakeholders by having graduate students who were interested in our course provide insights during a virtual co-creation session. Three graduate students participated in the same activity as the instructional team via Zoom, using a shared Google Slides deck to create a concept for the course with the options laid out for each decision point. See Figure 4 for an example of one of the course concepts a graduate student put forth. All three students created a course concept in which community members have multiple roles, the final assignment is one presentation and one product, multiple groups bring design thinking critiques to class, and assignments receive feedback from teaching team and peers. Only the latter two of these overlapped with the consensus met by the research team. The co-creation session showed us what was important to students, which helped the research team to check their assumptions about student needs and make changes to key elements of the course.

Using these insights, the instructional team determined what to prototype by identifying six essential components and then conducted a rapid prototyping session for these components. We used Google Slides to create mock-ups of the learning objectives, the reflection process, the process for giving and receiving feedback, and structure of the class period. This rapid prototyping made ideas for each of these course elements tangible and helped us to see where decision points for each element were embedded. Only the process for giving and receiving feedback contained decision points that could all be resolved by the teaching team based on insights already generated by stakeholders. For other decision points, we determined that the best way forward was to ask for feedback from graduate students (*n* = 19) using a Google Form. For a longer description of the methods and findings (see Appendix A).

These inspiration and ideation activities directly informed the development of our course objectives, the course assignments, the role of reflection, the course instruction methods, the role of community partners, and the way we provided feedback. All of this was reflected in our syllabus (Appendix B). The course was taught for the first time in Spring 2021 as we transitioned to the implementation phase of IDEO.org's design thinking process.

## Discussion on the Practical Implications, Objectives and Lessons Learned

The method of explicitly using the design thinking process to engage stakeholders in the creation of an interdisciplinary design thinking course offers key insights that are generalizable to course design efforts regardless of area of study, level of training, or format (in-person vs. remote). This process can be replicated or adapted by other instructional teams. We recommend that those who engage in this process do so as a team, with co-instructors, teaching assistants, or other colleagues. We also recommend holding regular meetings with the instructional team to allow for the free flow of ideas, and accountability to the process and the stakeholders. This process is especially helpful for instructional teams who desire student engagement in the classroom because it involves stakeholders from the beginning, ensuring their articulated and unarticulated needs are met. Further, explicitly using IDEO.org's design thinking methods demystifies the course design process, making it more tangible, replicable, and open to constructive feedback from the students who participate in the process, students who learn about the process in the class, and the instructional team. We also recommend carefully documenting activities and decisions made for accountability and to ensure that stakeholders' insights are being captured and used to design the course. While the process took our team about a semester to complete, the individual activities we detailed did not take long. Instructional teams can incorporate a few design thinking activities into their engagement with stakeholders during course design, spending as much time as they have and need on each one. As we demonstrated, they can happen both virtually and in-person, so the instructional team does not need to gather for each activity. While the following lessons learned are especially relevant for courses that hope to teach innovation mindsets and skills for changemakers, the process that yielded these insights and lessons learned can be used broadly and repeated.

This course design process yielded five recommended practices for teaching design thinking specifically:

1. Design thinking pedagogy should be interdisciplinary when possible. This begins to break down institutional barriers and silos. It also meets a student desire to work on and learn from interdisciplinary teams.
2. Mindsets are just as important, if not more important, than methods. To develop mindsets and provide students with an opportunity to understand their own place in design thinking, courses should build in structured and regular reflection at consistent touchpoints in the course.
3. Skill building should be explicit, clearly structured and taught in a scaffolded way. Students want structure and scaffolding, with opportunities to dive deeper on topics and methods they have experience with. As they develop these skills that transcend disciplines, students want feedback more than actual grades and want to work with intentionally designed groups. Further, design thinking should be situated among other problem-solving models and methods, so that students may better connect design thinking to what they already know.
4. Students who are learning and applying complicated methods for the first time are not the best positioned to serve as consultants for community partners. Instructors who want to incorporate community partnerships must find a way to balance the tension between wanting students to learn the methods and wanting students to apply the content to real-world projects. This can be achieved through intentional and meaningful partnerships with community partners that benefit both parties.

We have presented a detailed description of our course design process for a new interdisciplinary design thinking course, with key insights from the inspiration and ideation stages of the design process. The instructional team worked alongside key stakeholder groups to develop the course, iterating, and gaining feedback along the way. In detailing our method, we provide a means for course design regardless of area of study, level of training, or format (in-person vs. remote). The nature of our course yields insights and strategies that are especially relevant for the design of courses that teach innovation and design methods to interdisciplinary teams of students. Our design work has also yielded key insights relevant to the teaching of design thinking courses specifically. We hope we inspire instructional teams to intentionally build future courses using a similar process. Our next step is to conduct a follow-up study on our first pilot after the Spring 2021 semester.

## Acknowledgement of Any Conceptual, Methodological, Environmental, or Material Constraints

A limitation of this work is that community partners were not included as a key stakeholder group; we did not set out to design a community-engaged course so did not recruit community partners in our inspiration or ideation phases. We recommend that any future work that replicates or adapts this process makes room for the inclusion of additional stakeholders as their role becomes clear. We also want to acknowledge that this process was fairly time intensive. Over the course of the semester we spent 10–15 h per week on this project. We understand that not all instructional teams will be able to dedicate this amount of time to developing a new course or updating an existing course but still recommend that they take aspects of this course design process (phases, activities, etc.) and integrate them into their own process. While we plan to publish the results of our Spring 2021 implementation of the course and present evaluation outcomes related to changes in mindsets and design thinking skills, we are confident that this investment of time and resource into this course development project will yield valuable and beneficial outcomes for all stakeholders involved.

## Data Availability Statement

The original contributions presented in the study are included in the article/Supplementary Material, further inquiries can be directed to the corresponding author/s.

## Author Contributions

ES, EC, and VJ contributed to the design and implementation of the research, to the analysis of the results, and to the writing of the manuscript. All authors contributed to the article and approved the submitted version.

## Conflict of Interest

The authors declare that the research was conducted in the absence of any commercial or financial relationships that could be construed as a potential conflict of interest.

## Publisher's Note

All claims expressed in this article are solely those of the authors and do not necessarily represent those of their affiliated organizations, or those of the publisher, the editors and the reviewers. Any product that may be evaluated in this article, or claim that may be made by its manufacturer, is not guaranteed or endorsed by the publisher.
