# Supplementary material for: Using the Design Thinking Process to Co-create a New, Interdisciplinary Design Thinking Course to Train 21st Century Graduate Students
Source: Front Public Health. 2022 Jan 11;9:777869. doi: 10.3389/fpubh.2021.777869 (PMC8802717; doi:10.3389/fpubh.2021.777869)
Supplement: Supplementary file 1 [file Table_1.docx]

**Appendix A**

| **Phase** | **IDEO.org Method** | **IDEO.org Activity Description (IDEO.org, 2015)** | **Research Team Process** | **Questions/Insights Generated** |
| --- | --- | --- | --- | --- |
| Inspiration | Frame Your Design Challenge | Getting the right frame on your design challenge will get you off on the right foot, organize how you think about your solution, and at moments of ambiguity, help clarify where you should push your design. Framing your design challenge is more art than science, but there are a few key things to keep in mind. First, ask yourself: Does my challenge drive toward ultimate impact, allow for a variety of solutions, and take into account context? Dial those in, and then refine it until it’s the challenge you’re excited to tackle.  Steps:   1. Start by taking a first stab at writing your design challenge. It should be short and easy to remember, a single sentence that conveys what you want to do. We often phrase these as questions which set you and your team up to be solution-oriented and to generate lots of ideas along the way. 2. Properly framed design challenges drive toward ultimate impact, allow for a variety of solutions, and take into account constraints and context. Now try articulating it again with those factors in mind. 3. Another common pitfall when scoping a design challenge is going either too narrow or too broad. A narrowly scoped challenge won’t offer enough room to explore creative solutions. And a broadly scoped challenge won’t give you any idea where to start. 4. Now that you’ve run your challenge through these filters, do it again. It may seem repetitive, but the right question is key to arriving at a good solution. A quick test we often run on a design challenge is to see if we can come up with five possible solutions in just a few minutes. If so, you’re likely on the right track. | Each team member independently completed a Frame Your Design Challenge worksheet, which allowed for discussion of three how might we (HMW) statements. This discussion included creating an updated HMW statement as well as brainstorming possible solutions, constraints, and methods for research. | Our guiding HMW statement, “How might we recruit and equip an interdisciplinary team of UNC-CH grad students to apply design thinking approaches, tools, and mindsets to solve diverse and complex problems and share their process and insights with community members?” was developed. Potential solutions including an accelerator model or competition where students get funding for projects, a train-the-teacher model, a service-learning model, project sourced by the university, access to people who are already design thinking practitioners, realistic but controlled case study or a virtual course were all discussed. Potential constraints including the fixed amount of time allowed by a 3-credit course, funding, the need to assess/evaluate students individually, student mindsets, access to users, and student engagement were identified. Research methods including speaking to researchers at other universities who are teaching design thinking well, participating in an analogous experience, and reviewing existing design thinking syllabi were determined. |
| Inspiration | Build A Team | Human-centered design works best with cross-disciplinary teams. You could put three business designers to work on a new social enterprise, but if you throw a graphic designer, a journalist, or an industrial designer into the mix, you’re going to bring new modes of thinking to your team. It’s smart to have a hunch about what kind of talent your team will need—if you are designing a social enterprise, a business designer is probably a good bet—but you won’t get unexpected solutions with an expected team.  Steps:   1. First, assess how many team members you’ll need, your staff’s availability, and when your project should start and end. 2. Look at the core members of your team and determine what they’re good at and what they’re not so good at. 3. Is there a clear technical capability that you’ll need but don’t currently have—maybe a mechanical engineer, a graphic designer, a skilled writer? Remember that you can always add a team member for a shorter period of time when their skills are most important. | The ideal team size was determined to be three to allow for ease of communication and the free movement of ideas. Dr. Chen (Design Thinking Lead at Innovate Carolina) Ms. Jagannathan (Co-Founder of Rural Opportunity Institute) both brought to the team IDEO design thinking training, experience applying the design thinking process to the creation of a tech non-profit and experience teaching. Dr. Chen brought additional teaching experience at the graduate level. Ms. Jagannathan had additional business and start-up experience. Ms. Skywark joined the team as a Graduate Research Assistant who brought a graduate student perspective and qualitative research training. | We found that having a team with similar experiences, but who functioned in different roles, allowed for a depth of insights that could not have been gleaned if all team members designing this course had been graduate-level professors. |
| Inspiration | Recruiting Tools | Before you start talking to the people you’re designing for, it’s important to have a strategy around who you talk to, what you ask them, and what pieces of information you need to gather. By planning ahead and tracking who you talk to once you’ve done it, you can be sure to have the right balance of experts and laymen, women and men, people of different ethnicities and classes, as well as a full range of behaviors, beliefs, and perspectives.  STEPS   1. Refer to [Extremes and Mainstreams](http://www.designkit.org/methods/45) to make sure that you’re talking to a broad spectrum of people. 2. As you determine who you want to talk to, think about a variety of factors: age, gender, ethnicity, class, social position. 3. Be sensitive to gender when making your interview plan. Some communities may not be comfortable with men interviewing women. Or if you’re working on a touchy topic, like open defecation, make sure that you understand social dynamics before you begin your Interviews. 4. [Group Interviews](http://www.designkit.org/methods/20) can be a highly useful tool and also help you identify who you might like to speak more with in an individual interview. | We knew we needed generate insights from interviewing graduate students. To maximize resources, we decided to speak only to graduate students who showed interest in taking the course. Recruitment materials were created with this specific group as the target. | A digital poster was created, advertising for the new course with a QR code that linked to a brief survey. This poster was distributed through university listservs and posted in targeting buildings. It was effective at soliciting 43 survey responses from potential users. A permission form, which explained the team’s research plan and objectives, was distributed to students selected for interviews. These effective recruitment tools also served as a pilot of our marketing plan for the course. |
| Inspiration | Secondary Research | Human-centered design is all about talking with people about their challenges, ambitions, and constraints. But as you move through the Inspiration Phase there will be moments where you’ll need more context, history, or data than a man-on-the-street style Interview can afford. Social sector challenges can be really thorny, which is why Secondary Research, whether done online, by reading books, or by crunching numbers, can help you ask the right questions. At [IDEO.org](http://www.ideo.org), we find time and again that a firm foundation of knowledge is the best place from which to tackle a design challenge.  STEPS   1. Once you know your design challenge, it’s time to start learning about its broader context. You can bone up quickly by exploring the most recent news in the field. Use the Internet, newspapers, magazines, or journals to know what’s new. 2. Try to find recent innovations in your particular area. They could be technological, behavioral, or cultural. Understanding the edge of what’s possible will help you ask great questions. 3. Take a look at other solutions in your area. Which ones worked? Which ones didn’t? Are there any that feel similar to what you might design? Any solutions that have inspired you to make one of your own? 4. Because interviews can be highly qualitative, use your Secondary Research to get the facts and figures you need to understand the context of your challenge. | We gathered a collection of 16 articles design thinking training/research and 14 articles about design thinking curricula/pedagogy. Together we reviewed the 13 most relevant articles, seeking definitions for social innovation and design thinking, paying attention to audience, synthesizing main takeaways, and generating new ideas/questions for our course. | - Should design thinking education include a review of design thinking’s history? When during the course should students be exposed to critiques of design thinking? - How might we teach the many applications of design thinking, beyond consumer-facing interventions or business? - How might we effectively use illustrative case studies? How will we gather these case studies? What design thinking examples will be most relevant for students in our course? How do we figure this out? - What do we want our students to feel/experience because of their course participation? How can we replicate the extreme circumstances of wicked problems in case studies for students? - Students are interested in working on projects they are interested in. How can we take this deeper than content level interests and closer to personal values/goals level interests? - How might we break down the divide between knowledge acquisition and knowledge use? - What is the role of community engagement? - What is our broader goal beyond students applying and communicating about design thinking? What topical knowledge, if any, do we want to assess? - How often do we want to do full design sprints? - How much implementation/evaluation do we want to build into the course? - Do we want to teach design thinking as is -- or a different flavor of design thinking that is more participatory? - How will we meaningfully build divergent thinking skills? How will we strengthen systems thinking? How would we assess students' ability to master divergent thinking or systems thinking? |
| Inspiration | Expert Interviews | Though the crux of Inspiration phase is talking with the people you’re designing for, you can gain valuable perspective by talking to experts. Experts can often give you a systems-level view of your project area, tell you about recent innovations—successes and failures—and offer the perspectives of organizations like banks, governments, or NGOs. You might also look to experts for specific technical advice.  STEPS   1. Determine what kind of expert you need. If you’re working in agriculture, perhaps an agronomist. In reproductive health? A doctor or policymaker may be a good bet. 2. When recruiting your experts, give them a preview of the kinds of questions you’ll be asking and let them know how much of their time you’ll need. 3. Choose experts with varying points of view. You don’t want the same opinions over and over. 4. Ask smart, researched questions. Though you should come prepared with an idea of what you’d like to learn, make sure your game plan is flexible enough to allow you to pursue unexpected lines of inquiry. 5. Record your Interview with whatever tools you have. A pen and paper work fine. | A list of UNC-CH faculty who teach design thinking was collected. These faculty members were introduced to the project and asked to complete a survey about their experience being trained in and teaching design thinking. Of the 13 faculty contacted, 11 responded to the survey. Five were selected for interviews and the following questions were asked of them:   1. You indicated on your survey that you learned/got trained in design thinking through [insert response]. Please tell us more about how you learned about DT. 2. What did you like about the way you learned about DT? 3. What did you dislike about the way you learned about DT? 4. How would you have preferred to learn about DT? 5. Why did you start teaching design thinking? 6. How did you choose the version of design thinking that you teach? 7. What’s your favorite part of teaching this course? 8. What’s the hardest part of teaching this course? 9. How has your DT course changed over time? 10. What are the student outcomes you hope to achieve through teaching your course? How do you measure that? 11. If you could do anything with this course to improve this course (the sky was the limit), what would you do and why? | - Design thinking is being practiced and taught across the university; it is not isolated to any one school, department, or discipline. Design thinking is interdisciplinary and has many applications. - There is no one way to be trained in design thinking. Our design thinking faculty have used almost every available method for obtaining formal and informal design thinking training. IDEO and the Stanford d.School methods of design thinking are practiced most frequently (~60%) at UNC-CH. The distribution of methods practiced by faculty closely aligns with the methods taught by faculty. However, some faculty create modified or hybrid versions for the classroom. - Almost all faculty’s hopes for students after taking design thinking courses are related to mindset. This developed mindset will allow students to empathize, solve complex problems, be creative, etc. throughout their lives. Reflection aids in the development and measurement of these mindsets. - Teaching design thinking is hard, and the classroom environment matters. Students need a gradual release, as design thinking is new and unfamiliar at first, as well as a scaffolding of skills. Across courses, the empathy stage of the design thinking process is both the most labor intensive and the most important. - How can we measure changing student mindsets across the university with regards to design thinking? - How might we collaborate across schools/departments to strengthen students’ learning and application of design thinking? |
| Inspiration | Extremes and Mainstreams | When recruiting people to interview, target both the big broad mainstream and those on either extreme of the spectrum. An idea that suits an extreme user will nearly certainly work for the majority of others. And without understanding what people on the far reaches of your solution need, you’ll never arrive at solutions that can work for everyone. More importantly, talking to Extreme users can spark your creativity by exposing you to use cases, hacks, and design opportunities that you’d never have imagined.  STEPS   1. Think about all the different people who might use your solution. Extreme users can fall on a number of spectrums, and you’ll want variety. Maybe you’ll want to talk to someone who lives alone and someone who lives with a large extended family. Maybe you’ll want to talk to both the elderly and children. Each will offer a take on your idea that can spur new thinking. 2. When you talk to an extreme, ask them how they would use your solution. Ask them if they use something similar now and how it does or does not suit their needs. 3. Select appropriate community contacts to help arrange meetings and individual Interviews. Make sure you’re talking to men and women. You might even stumble across an extreme user in another context and want to talk to them there. 4. Be sensitive to certain extremes when you Interview them. They may often be left out of discussions like these so make them feel welcome and let them know that their voices are critical to your research. | Of the 43 students who showed interest in our course by taking a survey, nine graduate students were interviewed. The students selected were determined by their program, school, and design thinking experience. A range of programs and schools was selected for diversity of backgrounds. Students with no design thinking experience were selected as the mainstreams and students with design thinking experience were selected as the extremes. They were asked the following questions:   1. How did you first hear about design thinking? 2. Design thinking is a creative problem-solving process that provides structure to lead to innovative solutions. How do you see yourself applying this to your chosen career goals/field? 3. How do you define social innovation? 4. What does “public good” mean? 5. How do you like to learn new, difficult concepts/processes? 6. What’s their favorite course they’ve taken? Why was this course their favorite? 7. What would be an effective way for us to market this new course? | Students are attracted to design thinking because of its non-traditional problem-solving approach. Though design thinking is new and unfamiliar at first, students seem to find an anchoring point through which they connect to design thinking. We saw this in connections made to similar creative processes and in the frequency with which Henry Ford’s faster horses quote was cited. From their courses, students want explicit skill building, especially communication skills, group work skills, and skills that prepare them for the workforce. Most courses that teach design thinking incorporate this skill development into some form of a team project. |
| Inspiration | Immersion | The Inspiration Phase is dedicated to hearing the voices and understanding the lives of the people you’re designing for. The best route to gaining that understanding is to talk to them in person, where they live, work, and lead their lives. Once you’re in-context there are lots of ways to observe the people you’re designing for. Spend a day shadowing them, have them walk you through how they make decisions, play fly on the wall, and observe them as they cook, socialize, visit the doctor—whatever is relevant to your challenge.  STEPS   1. As you Create a Project Plan, budget enough time and money to send team members into the field to spend time with the people you’re designing for. Try to organize a homestay if possible. 2. Once you’re there, observe as much as you can. It’s crucial to record exactly what you see and hear. It’s easy to interpret what’s in front of you before you’ve fully understood it, so be sure you’re taking down concrete details and quotes alongside your impressions. 3. A great Immersion technique is to shadow a person you’re designing for a day. Ask them all about their lives, how they make decisions, watch them socialize, work, and relax. 4. If you’ve got a shorter window for Immersion, you can still learn a lot by following someone for a few hours. Pay close attention to the person’s surroundings. You can learn a lot from them. | Two members of the research observed two design thinking courses (BUSI 521: Design Thinking and MEJO 463: News Lab) at UNC During both immersion experiences, the team had guiding questions. They used these guided questions as a reference during observation. Time-stamped description of the class period included notes about student behavior, engagement, and course content. Conversations with both professors provided additional insight. | - How explicit will we be in tying in versions of design thinking during lecture? - What is the role of laptops/technology in class? - How do we want to encourage broad participation and engagement? - Do we want to use the course as an opportunity for students to develop an innovation they may see through beyond the course? - How much time do we want to dedicate to lecture vs. group work? - What is the optimal group size? The groups seem to be 4-6 folks per team. - Do we want to create a course page to recruit interested students? - How might we provide easy access to users/customers for students to do interviews? - Memorable story: “It felt like molasses” teaching in the basement. Natural light and environment matters. - Memorable story: Team formation by picking four people you do not know to explicitly build skills getting to know and work with new people. - How often do we want to do design sprints? How can these sprints help students to scaffold skills? - Reflection is a key part of skill building. What role does reflection have in our course? - What opportunities do icebreakers provide and how often/when should these be used? |
| Inspiration | Analogous Inspiration | [IDEO.org](http://www.ideo.org) teams are often led by their intuition to take creative leaps. It may feel silly to visit an Apple store when you’re designing for those living in difficult circumstances, but you may unlock the key to a memorable customer experience or a compelling way to arrange products. Analogous Settings can help you isolate elements of an experience, interaction, or product, and then apply them to whatever design challenge you’re working on. Besides, getting out from behind your desk and into a new situation is always a great way to spur creative thinking.  STEPS   1. On a large sheet of paper, list the distinct activities, behaviors, and emotions you’re looking to research. 2. Next to each one, write down a setting or situation where you might observe this activity, behavior, or emotion. For example, if the activity is “use a device at the same time every day,” parallel situations might be how people use alarm clocks. 3. Have the team vote on the site visits that they would like to observe for inspiration and arrange for an observation visit. 4. When you make your visit, pay close attention to what it is you’re seeking to understand, but remain open to all kinds of other inspiration. | The research team participated in an escape room experience together. This experience was intentionally chosen because it is an activity that is non-liner. The team entered with guiding questions, and paid attention to how they felt throughout the experience. A detailed debrief followed the activity. | - - - - Do we want different teams or one team throughout the semester?       - How can we ensure fidelity of information delivered to teams?         - We liked the video at the beginning. It ensured that all teams started with the same message. We might want to use videos or other methods to keep information shared across everyone in course equal.         - The cheat sheet also ensured that all teams got the same information in hints.         - Like the methods of the cheat sheet and the video, we may want to pick tools for ourselves to use as reference points when students ask questions, so we are all referring to the same content.       - Where do we want to hold class?         - The theme allowed for buy-in. This could be analogous to a project that uses design thinking but has one theme.         - Designing is a non-traditional mindset, and you need to create a space that mimics a designer’s environment. This space needs to be inclusive and empowering. We will need to be intentional in how we market design thinking as something anyone can do.         - The physical space matters: people are willing to try something new in a new context.       - Do we want to have a “level-up” option for those experienced in design thinking?         - We liked that there were levels to the room.         - We may want to do levels of DT course with a baseline curriculum then extensions for challenges and learning for teams that have mastered the baseline. |
| Ideation | Download Your Learnings | Now that you’ve got a huge amount of notes, photos, impressions, and quotes, it’s time to start making sense of them. Because teamwork is so critical to human-centered design, [IDEO.org](http://www.ideo.org/) teams download their learnings as groups. One by one you’ll go around the room, capture your ideas and stories on Post-its, and put them on big sheets of paper. It’s critical to pay close attention to your teammates stories, learnings, and hunches. This is a rich and powerful way to share what you’ve heard and part of the goal is make your individual learnings part of the collective group’s knowledge base.  STEPS   1. Take turns Downloading. Start by getting rid of other distractions and sitting in a circle. 2. When it’s your turn, put all key information you’re about to share on Post-its and use them as you describe who you met, what you saw, the facts you gathered, and your impressions of the experience. 3. Cluster the Post-its together as you put them on the wall or on a board so that you have a record of your discussion. 4. When it’s not your turn, pay close attention. Feel free to ask questions if something isn’t clear. 5. This process is best done the day of an interview or after a day in the field. Download while your experiences and perceptions are fresh. | After interviews and experiences, team members downloaded learnings to an online Miro board. Each week, the team met virtually to discuss learnings, using the board as a guide and discussion tool. Post-its were clustered into six categories: about person/experience, memorable quotes, memorable stories, pain points, solutions/opportunities, ideas generated by person/experience. Post-its were color-coordinated with each color matched to a single person/experience.  Team members took turns sharing their post-its. | The team created three sets of Miro boards with hundreds of Post-It notes. Reviewing these boards weekly allowed for the team to iterate interview guides given new insights. This iteration strengthened insights generated from learnings and the creation of themes in later ideation activities. |
| Ideation | Find Themes | Once you’ve had a chance to [Download Your Learnings](http://www.designkit.org/methods/12) and [Share Inspiring Stories](http://www.designkit.org/methods/13), you’re ready to Find Themes. Take a good long look across your interviews, Analogous Inspiration, and other learnings. Have any patterns emerged? Is there a compelling insight you heard again and again? A consistent problem the people you’re designing for face? What feels significant? What surprised you? These themes are bound to change, but as you move through the Ideation phase, continue looking for Themes and sorting out what they mean.  STEPS   1. Gather your team around the Post-its. Move the most compelling, common, and inspiring quotes, stories, or ideas to a new board and sort them into categories. 2. Look for patterns and relationships between your categories and move the Post-its around as you continue grouping. The goal is to identify key Themes and then to translate them into opportunities for design. 3. Arrange and rearrange the Post-its, discuss, debate, and talk through what’s emerging. Don’t stop until everyone is satisfied that the clusters represent rich opportunities for design. 4. Identifying these Themes will help you [Create Frameworks](http://www.designkit.org/methods/14) and write [Design Principles](http://www.designkit.org/methods/27). | After downloading our learnings, the team made a second copy of each Miro board from which we could move Post-its into themes. We made large Post-its that represented major themes and clustered Post-its around these themes as a team. We did this in three waves to take advantage of inspiration after each download. | The following themes emerged when insights from interviews, analogous experiences and other learnings were bundled.   1. Students want and need structure. 2. Students want explicit skill building. These skills include group work skills, communication skills, and skills that prepare them for the workforce. 3. Students need a growth mindset. 4. Students should have the opportunity to “level-up”. 5. Students should learn by doing. 6. Students want a non-traditional problem-solving approach. 7. Students should not reinvent the wheel. A final project should be a learning opportunity and stop there. 8. Most courses, including design thinking courses, have a group work component. Team formation and group work is complicated. There are many ways to form teams and many expectations for group work. 9. Empathy is a key stage of the design process. It is often the most arduous and time consuming. 10. Students should reflect. Students should examine their own lenses/positions/power and should consider ethics. 11. Design thinking education should have a gradual release and/or scaffolding. 12. The classroom environment, both physically and emotionally, matters. 13. Students/others compare and contrast HCD with other approaches they know. 14. Design thinking can be biased toward certain learning styles. 15. Design thinking should be community-driven which can be messy. 16. Teaching design thinking is hard. 17. Design thinking is new and unfamiliar at first. Students struggle executing design thinking. 18. Design thinking outcomes are hard to measure. 19. Systems and structures need to change to support design thinking. |
| Ideation | Create Insight Statements | You’ve heard a lot from a lot of different people, downloaded learnings, and identified key themes from your research. The next step in the synthesis process is to Create Insight Statements, succinct sentences that will point the way forward. Insight statements are incredibly valuable as they’ll help you frame [How Might We](http://www.designkit.org/methods/3) questions and give shape and form to subsequent [Brainstorms](http://www.designkit.org/methods/1). It’s not always easy to create them, and it will probably take some work editing them down to the three to five main insights that will help you drive toward solutions.  STEPS   1. Take the themes that you identified in [Find Themes](http://www.designkit.org/methods/5) and put them up on a wall or board. 2. Now, take one of the themes and rephrase it as a short statement. You’re not looking for a solution here, merely transforming a theme into what feels like a core insight of your research. This is a building block, not a resolved question. 3. Once you’ve done this for all the themes, look back at your original design challenge. Sift through your insight statements and discard the ones that don’t directly relate to your challenge. You only want three to five insights statements. 4. Take another pass at refining your insights. Make sure that they convey the sense of a new perspective or possibility. Consider inviting someone who is not part of your team to read your insight statements and see how they resonate. | Ahead of a team meeting, each team member used identified themes to construct their own insight statements. These insight statements were then shared out with the whole team. Statements not related to the challenge were discarded. Similar statements were synthesized. This process allowed the team to come to consensus about project insight statements. | The following insight statements were discussed by the team:   - 1. Faculty and students want to situate design thinking among other problem-solving approaches (history of design thinking, complementary approaches, pros and cons, relationship with CBPR and community).   2. Faculty and students want the course to be appropriately scaffolded (learning for the sake of learning followed by learning to solve an external problem, using design thinking to improve an existing solution then using design thinking to create a brand-new solution).   3. Faculty and students want students to be able to apply design thinking on their own and communicate their process to others.   4. Students want a structured professional development opportunity to explicitly build skills (e.g., communication, group work).   5. Faculty want students to reflect in the course (e.g., lenses, biases, ethics, power, positionality) and build integral mindsets (e.g., growth mindset, empathy).   The following insight statements related to points of tension that may lead to design opportunities were identified by the team.   - 1. Students want to create a meaningful work product by the end of the course, but community partners often can’t tolerate mistakes and failure.   2. Design thinking is inherently cross-disciplinary, but university structures and habits are siloed by default.   3. Faculty (and the world) prefer that students work in interdisciplinary teams to optimally apply the design thinking process, but students often prefer to work alone or with like-minded peers.   4. Breadth vs. depth is in tension amidst semester time constraints. Students and faculty want participants both to master the process, and have meaningful time/space to reflect, but class time is limited. |
| Ideation | How Might We | By defining themes and insights, you’ve identified problem areas that pose challenges to the people you’re designing for. Now, try reframing your insight statements as How Might We questions to turn those challenges into opportunities for design. We use the How Might We format because it suggests that a solution is possible and because they offer you the chance to answer them in a variety of ways. A properly framed How Might We doesn’t suggest a particular solution, but gives you the perfect frame for innovative thinking.  STEPS   1. Start by looking at the insight statements that you’ve created. Try rephrasing them as questions by adding “How might we” at the beginning. 2. The goal is to find opportunities for design, so if your insights suggest several How Might We questions that’s great. 3. Now take a look at your How Might We question and ask yourself if it allows for a variety of solutions. If it doesn’t, broaden it. Your How Might We should generate a number of possible answers and will become a launchpad for your Brainstorms. 4. Finally, make sure that your How Might We’s aren’t too broad. It’s a tricky process but a good How Might We should give you both a narrow enough frame to let you know where to start your Brainstorm, but also enough breadth to give you room to explore wild ideas. | Ahead of a team meeting, each team member used themes and insight statements to independently construct new How Might We question. During a virtual team meeting, each team member shared their statements. Similar trends and points of tension emerged, which allowed for the construction of a set of five team HMW statements that would inform design opportunities. | The team developed the following HMW questions:   1. HMW situate design thinking among other problem-solving approaches throughout the course? 2. HMW provide students with real-life learning experiences, while ensuring that community partners’ expectations are met? 3. HMW effectively support and equip students to engage in interdisciplinary collaboration within traditional university environments? 4. HMW ensure that students experience mastery of the design process (scaffolded structure that has opportunities for leveling up, growth mindset) to a depth that they can apply in the future, while also creating space for adequate reflection (on lenses, biases, ethics, power, positionality)? 5. HMW create a learning environment that is both physically and emotionally inviting for all types of thinkers? |
| Ideation | Design Principles | Think of Design Principles as the guardrails of your solution—quick, memorable recipes that will help keep further iterations consistent. These principles describe the most important elements of your solution and give integrity and form to what you’re designing. Odds are, they will align with the [Themes](http://www.designkit.org/methods/5) you find during the Ideation Phase. You’ll also find that they’ll evolve as you design things, so don’t be afraid to revise them. Keep them short and memorable, like, “Talk like people talk,” “The service always connects to the community,” or “Keep women at the center of business.” Lower-level ideas like “The logo should be blue” or “We offer discounts” are not Design Principles.  STEPS   1. Look at your Post-its, and your Themes in particular. 2. Consider the core principles underpinning those themes. Frame these as positive statements that might tell you how and what to design. Remember, Design Principles operate as a group, and it’s likely that you’ll need to identify several. 3. Look at the Design Principles you’ve come up with. Are they short and to the point? Do they describe just one idea? Try to avoid overly complicating them. If it feels like there are multiple ideas going on, break them into smaller parts. 4. Review your Design Principles and make sure they cover the key aspects of your solution. Modify any that don’t.   Be ready to revise your Design Principles as you start to build Prototypes and test your ideas. Some Design Principles won’t reveal themselves until you’ve actually designed something, but once you spot them they’ll quickly become essential. | Positive insights related to each of the team’s five HMW questions were brainstormed during a virtual team meeting. These insights were then turned into 1-2 design principles for each HMW statement. | The following design principles were developed as a framework for the design challenge.   1. **We view design thinking as one tool in a problem-solving toolkit.** We will build on what students already know and methods they already use, show how design thinking fits in with other problem-solving approaches students are familiar with, compare/contrast what students already know with what design thinking is/isn’t, and remind students that design thinking isn’t nirvana. 2. **We ensure that students, community partners, and the teaching team mutually learn and benefit.** Everything that students do should be meaningful and purposeful. Students, community partners, and the teaching team co-create expectations together that evolve over time. 3. **We believe that group work is an art and a science.** More perspectives are better. Practice makes better; you can explicitly teach & grow group work skills. 4. **We view individual reflection as a part of the design process for mindset development.** Students will have to do (cognitive) work outside of class time. Reflection should be practiced and repeated. Skills should be scaffolded in course structure. 5. **We recognize that there are many types of learners in the classroom and create opportunities for all to thrive.** The physical learning environment should be bright and built for collaboration. The teaching team will model the growth mindset. 6. **We want 5-star reviews.** Students are our users/customers. The first version of HBEH 748 is a pilot in itself. 7. **We will be able to successfully deliver this course in-person or online.** |
| Ideation | Brainstorm | At IDEO.org, we use Brainstorms to tap into a broad body of knowledge and creativity. Over the course of your project you should do them not only with your design team, but also with partners and the people you're designing for. Refer to [Brainstorm Rules](http://www.designkit.org/methods/28) for the specifics of what make for a fruitful brainstorm, but remember that the best policy is to promote openness, lots of ideas, and creativity over immediate feasibility. Brainstorms work best when the group is positive, optimistic, and focused on generating as many ideas as possible.  STEPS   1. Pass out pens and Post-its to everyone and have a large piece of paper, wall, or whiteboard on which to stick them. 2. Review the Brainstorm Rules before you start. 3. Pose the question or prompt you want the group to answer. Even better if you write it down and post it. 4. As each person has an idea, have her describe to the group as she puts her Post-it on the wall or board. 5. Generate as many ideas as possible. | After five HMW statements were created, each team member independently brainstormed as many solutions as possible for each in five minutes. A Miro board was created with a section for each question. Team members added colored Post-it notes for each potential solution to each HMW section. The team then met virtually to share these ideas. As ideas were shared, team members clustered similar solutions together. | Through independent and team brainstorms 132 potential solutions were generated. |
| Ideation | Bundle Ideas | Bundling Ideas takes you from strong individual concepts to solutions of substance. Think of it as a game of mix and match, with the end goal of putting the best parts of several ideas together to create more complex concepts. You’ll probably notice that many ideas start to resemble each other—which is a good thing. Try combining them; keep the best parts of some, get rid of the ones that aren’t working, and consolidate your thinking into a few concepts you can now start to share with the world.  STEPS   1. You’ve got lots of drawings and ideas up on the wall, so now it’s time to start moving them around and forming them into more complex solutions. 2. Start by clustering similar ideas into groups. Talk about the best elements of those clusters and combine them with other clusters. 3. Now, start building groupings out of the [Themes](http://www.designkit.org/methods/5) and patterns you’ve found. Focus on translating what you’ve heard into practice, rather than just identifying similar ideas. 4. Once you’ve got a few idea groupings, ask yourself how the best elements of your thinking might live in a system. Now you’re moving from individual ideas to full-on solutions! | After the individual brainstorm and team debrief, ideas were bundled based on similarities in solutions. Solutions that the whole team had organically generated were bundled together into “things that will be true for our course”. Where there were different solutions, the team realized that decision points existed. Ideas related to these decision points were then bundled into eight decision point categories. | Things that will be true for our course:   1. We will explicitly provide group work tools 2. Building group work skills > creating awesome product 3. We will create opportunities for students to give/receive feedback on group work 4. We will strategically assign teams 5. Students should reflect on growth mindset, failure, and equity 6. Reflections will have structure and count toward grades 7. We will value community members’ time 8. We need to make accommodations for different learning styles and preferences 9. Physical space is important and should be inviting 10. The teaching team should be available and inviting   Decision Points:   1. What role will community members play in the course? 2. Who (students/teaching team/community members) will bring content about other problem-solving models and skills into the classroom? 3. Who (students/teaching team/community members) will bring critiques of design thinking into the classroom? 4. What is the percentage breakdown of group vs. individual work? 5. What is the nature of the final project? 6. What is the grading structure of the final project (in pieces throughout the semester or cumulatively at the end)? 7. What is the nature of individual assignments? 8. Who should deliver feedback on course assignments? |
| Ideation | Create a Concept | So far you’ve come up with, shared, and even discarded scores of ideas. You further refined things as you [Bundled Your Ideas](http://www.designkit.org/methods/30) and now it’s time to turn them into a Concept. A Concept is more polished and complete than an idea. It’s more sophisticated, it’s something that you’ll want to test with the people you’re designing for, and it’s starting to look like an answer to your [How Might We](http://www.designkit.org/methods/3) question. This is the moment where you move from problem to solution and it drives everything that comes next.  STEPS   1. Take the ideas that you bundled in [Bundle Your Ideas](http://www.designkit.org/methods/30) and put them up in the wall on Post-its. 2. Now might be a good time to [Create Frameworks](http://www.designkit.org/methods/14) out of those Bundled Ideas. Start to visualize where your Bundles are pointing, but think especially hard about making them into a system. 3. Don’t worry too much about all the details of your solution now—you don’t need a finely tuned funding strategy just yet. The goal is to get a robust, flexible Concept that addresses the problem you’re trying to solve. 4. Keep referring back to your [How Might We](http://www.designkit.org/methods/3) question. Are you answering it? Are there elements missing in your solution? What else can you incorporate to come up with a great solution? 5. Like the rest of human-centered design, there’s a bit of trial and error here. And Creating a Concept means you’ll probably create a couple that don’t work out. That’s fine. | All possible answers to decision point questions were brainstormed. Each team member independently created a concept for the course by selecting one option from each decision point. The team then compared their concept, looking for points of agreement. Where the team disagreed, each team member explained her rationale. | The team all agreed on where feedback should come from, who should bring critiques of design thinking to class, how the final project is graded and who should facilitate comparison to things students already know.  All Three Team Members Agreed:   - 1. Multiple groups should bring design thinking critiques to class   2. Assignments should receive feedback from the teaching team and peers   3. Parts of the final project should be graded along the way, allowing for iteration   4. Comparison to design thinking knowledge the students already know should be facilitated by students. |
| Ideation | Co-creation Session | You’ll be talking with loads of people over the course of your project, and a Co-Creation Session is a great way to get feedback on your ideas. The purpose of a Co-Creation Session is to convene a group of people you’re designing for and then bring them into the design process. You’re not just hearing their voices, you’re empowering them to make alongside you. You can co-create services, investigate how communities work, understand how to name your solution, or what its logo should look like. Not only is a community far more likely to adopt a practice or service that it helped create, but you’ll also gain valuable insight into all facets of your solution.  STEPS   1. The first step is to identify who you want in your Co-Creation Session. Perhaps it’s a handful of people you’ve already interviewed. Maybe it’s a particular demographic like teens or female farmers or people without jobs. 2. Once you know who you want, arrange a space, get the necessary supplies (often pens, Post-its, paper, maybe art supplies), and invite them to join. 3. Make the most of a Co-Creation Session with [Conversation Starters](http://www.designkit.org/methods/44), a [Brainstorm](http://www.designkit.org/methods/1), [Role Plays](http://www.designkit.org/methods/36), [Rapid Prototyping](http://www.designkit.org/methods/26/), or other activities to get your group engaged around the problem you’re looking to solve. 4. Capture the feedback your group gives you. The goal here isn’t just to hear from people, it’s to invite them into your design team. Make sure that you’re treating your Co-Creation as designers, not as interview subjects. | Graduate students who interviewed with the team during the inspiration phase were emailed a research update with a request to participate in a co-creation session to work through the eight decision points. Three graduate students participated in this session via Zoom. Two of these students study in the School of Education and one studies in the School of Dentistry. They participated in the same activity as the research team using a shared Google Slides deck, creating a concept for the course using options laid out for each decision point. The graduate students discussed their choices with the research team and with each other. Feedback on these decision points was captured. In remaining time, the graduate students also offered feedback that would inform what and how the teaching team chose to prototype. | All three graduate students included the following in their course concept:   1. Community members will have multiple roles 2. The final assignment should be one presentation and one product 3. Multiple groups bring design thinking critiques to class 4. Assignments receive feedback from teaching team and peers   Student feedback related to selections:  Graduate Student #1-   - Likes the ideas of community members having different roles like clients and mentors so they can share their different experiences. - Wanted parts of the final project to get graded along the way AND for the different parts to be graded at the end. That way, if someone got a 70 earlier, got feedback, and adjusted, they could raise that grade to an 85 at the end. - Believes it’s good for the final project to have a presentation because “communicating it out is important.”   Graduate Student #2:   - Expressed that it’s the Teaching Team (TT)’s responsibility to provide compare/contrast content because only the TT “will know where students are coming from.” - Critiques should be offered by TT and students: “Over the course, we could get there…gradual release. We need it to be facilitated though.” She wanted the TT to offer critiques first and said that it would be natural if students then started to offer their own critiques the more, they learned in the course. - Would expect that the first half of the course would focus on individual knowledge and skill development as well as reflection and then transition to more group work in the second half of the course - Picked both a final presentation and a final product because it’s important for students to communicate and write. - Liked having “check-ins” with members of the TT and emphasized that feedback on her work is more important to her than the grade itself. That being said, she was wondering if the course could be pass/fail.   Graduate Student #3:   - Wants TT to present comparative content on DT and other methods. She said that if she was expected to do this that she “would feel lost”, especially at the start of the course. She said she would “prefer if it were laid out” because she didn’t want to miss key content. - Wanted a final presentation and final product because it was “well-rounded.” - Liked the ideas of having check-ins with the TT throughout the final project. She wanted the bigger project broken down into smaller pieces. |
| Ideation | Determine What to Prototype | Your idea will have lots of testable components, so be clear about what you need to learn and which components will give you the necessary answers. Prototyping isn’t about being precious. Make simple, scrappy prototypes to not only save time, but to focus testing on just the critical elements. You might be trying to learn something like, “How big should this be?” or “What should the uniforms of the social enterprise look like?” At this stage you should have a lot of questions about how your idea should work. This is a great way to begin answering them.  STEPS   1. With your team, write down the key elements of your idea. Think practically about what needs to be tested and write down your primary questions for each component. 2. Now pick a few questions to answer. If you want to prototype an interaction, consider putting on a skit with your team. If you’re testing a logo, print it out and stick it on a t-shirt to solicit feedback. 3. Think through what kind of prototype makes the most sense to answer these questions. You might consider holding a [Brainstorm](http://www.designkit.org/methods/1) now. 4. Remember, this process is about learning, not getting it right the first time. Better to test a miserable failure and learn from it, rather than take ages making a beautiful, highly refined prototype. | Together the research team identified six components that needed to be prototyped. It was determined that learning objectives and a final project needed to be prototyped to bookend the course, allowing the team to understand where the course began and where it was headed. Key elements of the course also needed to be tested. These included the reflection process, the structure of the class period, the process of giving and receiving feedback, and recruitment materials. For each of these six elements, the team determined what needed to be prototyped, with whom it would need to be prototyped and how it should be prototyped. In this stage, it was determined that the final project and recruitment materials would need to be prototyped in a second wave to allow for iteration of elements that would affect these. | The following elements were selected for prototyping in certain forms and with particular groups.   - Learning objectives   - Prototype language for learning objectives   - Prototype with graduate students and Innovation for the Public Good lead   - Prototype by sending objectives with feedback form - Reflection process   - Prototype specific reflection activities   - Prototype with Faculty at UNC-CH and other institutions who emphasized the importance of reflection (Melissa, Danielle)   - Prototype via 1-hour Zoom call where we prototype 2 versions and solicit feedback (could send prototypes ahead of time and questions to consider) - Structure/agenda of a class period? (We are assuming that whatever structure we have needs to work for in-person and virtual learning)   - Prototype if structure is weekly and repeatable for 3-hour seminar (2-4 options)   - Prototype if part of the 3-hour seminar is structured and repeated (1-1.5 hours of the 3; other time could be unstructured) (2-4 options)   - Prototype if structure/agenda rotates every week (e.g., One week is group work, one week is case studies, one week is individual reflection, and repeat) (2+ options)   - Ideally, we’d prototype both in person and virtually, but we may be limited this spring w/ social distancing   - Prototype with UNC-CH faculty and graduate students   - Prototype via 1-hour Zoom call where we prototype 2 versions and solicit feedback (could send prototypes (potentially in the form of storyboards) ahead of time and questions to consider) - Process of giving/receiving peer feedback   - Prototype how peers are matched with other peers (They choose, TT assigns, it rotates, etc.)   - Prototype how feedback is delivered (In-person, virtually, hybrid)   - Prototype what kind of feedback is delivered (content, formatting/style, copyediting, big picture)   - Prototype if peer feedback graded or assessed, if at all?   - Prototype with grad students (the same group as reflections) and UNC-CH faculty team (the same group that we discuss w/ class structure/reflection)   - Prototype via 1-hour Zoom call where we prototype 2 versions and solicit feedback (Could send prototypes (potentially in the form of storyboards) ahead of time and questions to consider) - Recruitment materials   - Prototype course flyer and email to accompany course flyer   - Prototype with grad students (wider net of grad students beyond Google Form)   - Disseminate by emailing course flyer out to different schools and have grad students sign up for an email list and see which email/flyer combo let to the most sign ups - Final project   - Pause to prototype until community member role is known   - Prototype with graduate students, community members, faculty at UNC-CH who teach design thinking   - Share through 1-hour Zoom call where we prototype 2 versions and solicit feedback |
| Ideation | Rapid Prototyping | For human-centered designers, prototyping is an incredibly effective way to make ideas tangible, to learn through making, and to quickly get key feedback from the people you’re designing for. Rapid testing with real users can help you identify concepts that have potential for impact and spot ways to improve on early ideas. Prototyping isn’t about being precious. Simple, scrappy prototypes will not only save time, but also help focus testing on just the critical elements. The steps outlined below could take anything from a few days to a few weeks, depending on the challenge you’re solving. Follow them at a pace that works for you.  STEPS   1. After you have brainstormed ideas and bundled those ideas into concepts you’ll want to select a few of the most promising concepts to bring forward into testing. Use the [Determine What To Prototype](https://www.designkit.org/methods/determine-what-to-prototype) activity to help you select concepts and get clear on what you need to learn. 2. Once you’ve determined what to prototype, the Prototypes activity can help you find fast and scrappy ways to do your testing. This stage of the design process could take anything from a few days to a few weeks, depending on what you’re testing and how many rounds of prototyping you want to do. 3. You’ll probably be testing several prototypes at once, so you’ll need to capture your findings in an organized in order to learn about what works and decide what to iterate upon or take forward, using the [Prototype Report Card worksheet](https://design-kit-production.s3-us-west-1.amazonaws.com/Design+Kit+Method+Worksheets/DesignKit_prototypereportcard_worksheet.pdf). 4. Prototyping is an iterative process and so you may repeat this sequence several times over. Each time you will [Integrate feedback and Iterate](https://www.designkit.org/methods/integrate-feedback-and-iterate) upon your solution, to improve and refine it. And with each new round of testing your learning goals will become more tightly focused, as you make decisions about what to take forward. | The team created a [Google Forms survey](https://docs.google.com/forms/d/1LKVsog_8jvMUAolbSs_dfwabOBMfXUiL0Q257Co7OZU/edit?usp=drive_web) to gather feedback from a pool of graduate students interested in the course. The survey asked for feedback on the five course learning objectives, the format, timing and frequency of reflections, and the course structure. Nineteen graduate students responded.  For reference, the learning objectives presented to students were:  1. Identify how to center innovation designs as a response to the voice, experiences, wishes and aspirations of those most directly impacted by innovation.  2. Develop an understanding of one's own experiences, intentions, strengths and limitations, motivations, and biases as a change maker relative to the impacted audiences.  3. Identify, define, and clearly analyze a problem, recognize opportunities, challenges, and the assets of communities as they address the problem, and generate optimal solutions through the application of social innovation in practice.  4. Understand how the context in which a problem is located, and solution is imagined shapes and impacts the innovation design and implementation process.  5. Understanding how to effectively engage stakeholders in co-design, implementation, evaluation, and adaptive learning associated with the innovation. | **Learning Objectives**   - These learning objectives align with students’ future plans because they afford them the opportunity to develop a new perspective and provide skills for innovation with real-life application and working with stakeholders/community engagement/co-creating. - The most students (n=9) are excited about Learning Objective 3. - About a fifth of the students (21.1%) were excited about Learning Objective 1 and about a fifth (21.1%) were excited about Learning Objective 5. - Learning Objective 1 would be newest to the most students (n=8), closely followed by Learning Objective 5 (n=6). No students selected - Learning Objective 2 as the one they were most excited about nor as the learning objective that was the newest.   **Reflections**   - For each decision point for frequency, format and grading of reflections some students selected each option and some students indicated that they had no preference at all. Thus, while there is a determinable top response, there is also a tiered range with some interest for all potential options. - *Frequency:* Students would like to reflect after specific moments in the course. If reflections happened at a regular frequency, students would prefer this to be monthly. - *Format:* Almost all students (n=18) would be interested in submitting written reflections. About a quarter (n=5) of students would also be interested in audio reflections. - *Grading:* Most (n=11) students would like to receive feedback and a pass/fail grade for each reflection.   **Course Structure**   - *New Content:* When permitted to choose multiple options for how they would like to learn new content/concepts in this course, most students (n=16) would like community involvement with the first 1-hour of class spent with a guest speaker who shares new material, and many (n=9) would like traditional lecture with the first 1-hour of class spent covering new material/lecture. - *Design Challenges:* When asked how long each they would like a design challenge to be, no students selected one challenge for the entire semester nor one design challenge per week. Most students fell in the middle, selecting 3-4 design challenges across the semester (n=9) or one design challenge every 2-3 weeks (n=7). Those who selected neither of these had no preference (n=3). - *Week-to-Week Course Structure:* Most students (n=11) would prefer that the week-to-week course structure change, depending on course content and where we are in the semester. |

**Appendix B**

| 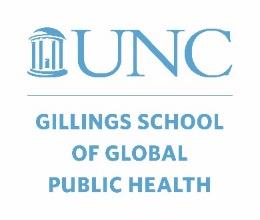 | **Syllabus**  HBEH 748: Design Thinking for the Public Good  Spring 2021  3 Credits \| Residential |
| --- | --- |

**Course Description**

This course will train an interdisciplinary group of graduate students to apply the mindsets, methods, and process associated with design thinking (i.e., human-centered design) to solve real world problems. Design thinking is a creative problem-solving process that prioritizes ethnographic market research, convergent and divergent thinking, as well as rapid prototyping. Students will collaborate with community members to design solutions (products, services, etc.) that are desirable, feasible, and viable.

**Prerequisites:** None. Registration priority will be given to students enrolled in the Innovation for the Public Good Graduate Certificate.

**Instructors**

Elizabeth Chen, PhD, MPH

Assistant Professor, Health Behavior MPH Concentration Lead

Department of Health Behavior

Vichi Jagannathan, MBA

Adjunct Professor

Department of Health Behavior

**Office Hours:**

Dr. Chen will host office hours on Thursday mornings via Zoom when she returns from family leave. To sign up for a 15-minute appointment, please schedule an appointment through Calendly.

Professor Jagannathan will host office hours on Mondays from 11:30am – 1:30pm via Zoom. To sign up for a 15-minute appointment, please schedule an appointment through Calendly.

**Course Website:**

Use your ONYEN and password.

**Class Days, Times, Location:** Tuesdays from 5:30-8:30 pm, Zoom (invites will be sent via Outlook calendar separately)

**Course Overview**

The overall goal of this course is to facilitate student application of the mindsets, methods, and process associated with design thinking (i.e., human-centered design) to solve real world problems.

By the end of the course, students will be able to independently:

- Identify how to center innovation designs as a response to the voice, experiences, wishes and aspirations of those most directly impacted by innovation
- Develop an understanding of one’s own experiences, intentions, strengths and limitations, motivations, and biases as a changemaker relative to the impacted audiences
- Identify, define, and clearly analyze a problem, recognize opportunities, challenges, and the assets of communities as they address the problem, and generate optimal solutions through the application of social innovation in practice
- Understand how the context in which a problem is located, and solution is imagined shapes and impacts the innovation design and implementation process
- Understand​ ​how​ ​to​ ​effectively​ ​engage​ ​stakeholders​ ​in co-​design, implementation, evaluation, and adaptive learning associated with the innovation

**Course Format**

The course will meet once per week for the duration of the semester. This course will be entirely remote. All class sessions will be recorded, and the recordings will be housed on Sakai.

Generally, each class session will consist of a lecture along with a combination of individual reflections, small group discussions, in-class exercises, case studies, examples from the design thinking literature, and time for group work. Students are expected to complete the readings before class and come to each class prepared to discuss the course materials. Asynchronously, students will be expected to complete written assignments and reflections.

**Course Materials**

All students should have a copy of the following materials:

1. The Field Guide to Human-Centered Design by IDEO.org
2. Field Guide: Equity-Centered Community Design by Creative Reaction Lab

**Community Norms**

We expect instructors, students, guest speakers, and others involved in HBEH 748 to adhere to the following community norms. These community norms come from the equity-centered nonprofit organization [Creative Reaction Lab](https://www.creativereactionlab.com/).

Our community norms include:

1. Be respectful of others – on their terms.
2. Acknowledge we’re all on a journey and part of the journey is growth.
3. Lean into discomfort.
4. Show up with humility and empathy even if opinions differ.
5. Be patient and hold yourself and others accountable to ultimately build trust with one another.
6. Address the issue, not the person.

We may add additional community norms to this list as the semester progresses, as needed.

**Enabling Videos on Zoom**

**We recommend that faculty and students enable videos during synchronous learning so that we can see each other.** Enabling videos will help instructors gauge student understanding and engagement so we can adjust instruction to better meet student needs.

We understand that students may prefer to disable their video if they need to minimize the activity on their internet connection. If this is the case, we ask that students continue to participate through the chat and other communications features in Zoom (e.g., raising hand, reactions for clapping or thumbs up).

We also recommend the use of Zoom backgrounds if that would facilitate students enabling their videos.

**Course-at-a-Glance**

Instructors reserve the right to make changes to the syllabus, including topics, readings, assignments, and due dates. Any changes will be announced as early as possible and noted on Sakai. For session-by-session course schedule details, please see the Sakai course site.

| **Date/ Session** | **Topic** | **Readings and Assignment Due** |
| --- | --- | --- |
| Class 1: Introduction (1/19) | Overview of design thinking  Ethical design thinking/critiques of design thinking | Required:   1. Read: Medium.com. [Racism and inequity are products of design. They can be redesigned.](https://medium.com/equity-design/racism-and-inequity-are-products-of-design-they-can-be-redesigned-12188363cc6a) 2. Read: Brown, T., & Wyatt, J. (2010). [Design thinking for social innovation.](https://ojs.unbc.ca/index.php/design/article/viewFile/1272/1089) Development Outreach, 12(1), 29-43. 3. Watch/Listen: [Critical and Emancipatory Design Thinking with Lesley-Ann Noel — DT101 E57](https://podcasts.google.com/feed/aHR0cHM6Ly9kZXNpZ250aGlua2luZzEwMS5saWJzeW4uY29tL3Jzcw/episode/ODE2MzNlNmEtZTEzMC00NzVmLThlNDMtNTQ3MDM4ZWFjMDQ3)   Recommended:   1. Read: Dorst, K. (2011). [The core of ‘design thinking’ and its application.](https://www.sciencedirect.com/science/article/pii/S0142694X11000603#!) Design studies, 32(6), 521-532. 2. Watch[: Justice by Design: A TED Talks About Equity, Community Design, and Living Experts](https://medium.com/equal-space/justice-by-design-a-ted-talks-about-equity-community-design-and-living-experts-bcc34778f3ab) 3. Listen: This is HCD. [Bas Raijmakers ‘How do we take a global perspective on HCD while respecting local differences’](https://www.thisishcd.com/episodes/bas-raijmakers-how-do-we-take-a-global-perspective-on-hcd-while-respecting-local-differences/). |
| Class 2: Inspiration (1/26) | Inspiration overview  [Starting your project,](https://www.designkit.org/methods/frame-your-design-challenge) [define your audience](https://www.designkit.org/methods/define-your-audience)[, frame your design challenge](https://www.designkit.org/methods/frame-your-design-challenge), [create a project plan](https://www.designkit.org/methods/create-a-project-plan), [build a team,](https://www.designkit.org/methods/build-a-team) [align your impact goals](https://www.designkit.org/methods/align-on-your-impact-goals), [recruiting tools](https://www.designkit.org/methods/recruiting-tools), [secondary research](https://www.designkit.org/methods/secondary-research), [immersion](https://www.designkit.org/methods/immersion), [analogous inspiration](https://www.designkit.org/methods/analogous-inspiration), [peers observing peers](https://www.designkit.org/methods/peers-observing-peers) | Required:   1. Read: Review methods for this week in *The Field Guide for Human-Centered Design* or the [Design Toolkit](https://www.ideo.com/post/design-kit) 2. Watch: IDEO.org. [Empathy.](https://www.designkit.org/mindsets/4) 3. Listen: [Problem Spaces, Understanding How People Think, and Practical Empathy with Indi Young — DT101 E6](https://fluidhive.com/problem-spaces-understanding-how-people-think-and-practical-empathy/)   Recommended:   1. Listen: IDEOU. [Why Leadership is Not About Having All the Answers](https://www.ideou.com/blogs/inspiration/why-leadership-is-not-about-having-all-the-answers). 2. Watch/Listen: [IDEO. (2013). A Lesson in Empathy.](https://designthinking.ideo.com/blog/a-lesson-in-empathy) 3. Watch/Listen: [TEDxSoMa - Dev Patnaik - Wired to Care: How Companies Prosper When They Create Widespread Empathy](https://www.youtube.com/watch?v=C1rF1id3nLo) |
| Class 3: Inspiration (2/2) | Engagement  [Extremes and mainstreams](https://www.designkit.org/methods/extremes-and-mainstreams), [interviews](https://www.designkit.org/methods/interview), [group interviews,](https://www.designkit.org/methods/group-interview) [expert interviews](https://www.designkit.org/methods/expert-interview), [conversation starters](https://www.designkit.org/methods/conversation-starters), [photojournal](https://www.designkit.org/methods/photojournal), [body language](https://www.designkit.org/methods/body-language), [the five whys](https://www.designkit.org/methods/the-five-whys), [card sort](https://www.designkit.org/methods/card-sort), [collage](https://www.designkit.org/methods/collage), [guided tour](https://www.designkit.org/methods/guided-tour), [draw it](https://www.designkit.org/methods/draw-it), [resource flow](https://www.designkit.org/methods/resource-flow) | Required:   1. Read: Review methods for this week in *The Field Guide for Human-Centered Design* or the [Design Toolkit](https://www.ideo.com/post/design-kit) 2. Watch/Listen: TBD   Recommended:   1. Peter Bregman. (2011). [How to Really Listen](https://hbr.org/2011/10/how-to-really-listen). 2. Listen: [Understanding Customers: Research, Insights, and Storytelling with Steve Portigal — DT101 E48](https://fluidhive.com/understanding-customers-research-insights-and-storytelling-with-steve-portigal-dt101-e48/) |
| Class 4: Inspiration (2/9) | Guest Lecture and Design Sprint #1  [Uncommon Commonalities](https://transformativetoolkit.org/activity/uncommon-commonalities) activity | Required:   1. Read: Center for Creative Leadership. [10 Steps for Establishing Team Norms.](https://www.ccl.org/articles/leading-effectively-articles/the-real-world-guide-to-team-norms/) 2. Read: Boudett, K. P., & Lockwood, M. (2019, July). [The Power of Team Norms.](http://www.ascd.org/publications/educational-leadership/jul19/vol76/num09/The-Power-of-Team-Norms.aspx) 3. Watch/Listen: [Valerie Alexander. TEDxPasadena. How to Outsmart Your Own Unconscious Bias.](https://www.ted.com/talks/valerie_alexander_how_to_outsmart_your_own_unconscious_bias)   Recommended:   1. Read: [6 Tips from IDEO Designers on How to Unlock Insightful Conversation](https://www.ideou.com/blogs/inspiration/6-tips-from-ideo-designers-on-how-to-unlock-insightful-conversation) |
| Class 5: Inspiration (2/23) | Group Project Intro: Guest lecture and team time (2 hours)  *Individual Assignment #1 due* | Required:   1. Read: Chamorro-Premuzic, E., Knight, R., Saunders, E., & Bukholtz, A. (2020, March 04). [How to Collaborate Effectively If Your Team Is Remote.](https://hbr.org/2018/02/how-to-collaborate-effectively-if-your-team-is-remote) 2. Watch/Listen: [Amy Edmondson: How to turn a group of strangers into a team.](https://www.ted.com/talks/amy_edmondson_how_to_turn_a_group_of_strangers_into_a_team)   Recommended:   1. Read: Derek Bok Center, Harvard University. [Group Work](https://bokcenter.harvard.edu/group-work). 2. Read: The New York Times. [How to Build a Successful Team](https://www.nytimes.com/guides/business/manage-a-successful-team). 3. Watch/Listen: Hello Monday Podcast. [Drafting Teams with Former NFL Talent Scout Michael Lombardi](https://podcasts.apple.com/de/podcast/drafting-teams-former-nfl-talent-scout-michael-lombardi/id1453893304?i=1000440441502&l=en) |
| Class 6: Ideation (3/2) | Ideation Day 1  [download your learnings](https://www.designkit.org/methods/download-your-learnings), [top five](https://www.designkit.org/methods/top-five), [find themes](https://www.designkit.org/methods/find-themes), [create insight statements](https://www.designkit.org/methods/create-insight-statements), [brainstorm](https://www.designkit.org/methods/brainstorm), [brainstorming rules](https://www.designkit.org/methods/brainstorm-rules), [get visual](https://www.designkit.org/methods/get-visual), [mash-ups](https://www.designkit.org/methods/mash-ups), [share inspiring stories](https://www.designkit.org/methods/share-inspiring-stories), [how might we](https://www.designkit.org/methods/how-might-we), [explore your hunch](https://www.designkit.org/methods/explore-your-hunch), [create frameworks](https://www.designkit.org/methods/create-frameworks), [bundle ideas,](https://www.designkit.org/methods/bundle-ideas) [design principles](https://www.designkit.org/methods/design-principles), [create a concept](https://www.designkit.org/methods/create-a-concept),  [Café without Coffee](https://transformativetoolkit.org/activity/cafe-without-coffee) activity  *Group Inspiration Report due* | Required:   1. Read: Review methods for this week in *The Field Guide for Human-Centered Design* or the [Design Toolkit](https://www.ideo.com/post/design-kit) 2. Watch/Listen: [Convergent v. Divergent Thinking. (2016). Harvard Professional Development.](https://www.youtube.com/watch?v=xjE2RV6IQzo) 3. Watch/Listen: [Divergent thinking in the 21st century \| Vihar Desu \| TEDxNewarkAcademy](https://www.youtube.com/watch?v=2FxMooLqC3A)   Recommended:   1. Read: [Strategies of Divergent Thinking](https://www.ccl.org/articles/leading-effectively-articles/the-real-world-guide-to-team-norms/) 2. Watch: IDEOU. [Divergent Thinking and the Innovation Funnel.](https://www.ideou.com/blogs/inspiration/brendan-boyle-on-divergent-thinking-and-the-innovation-funnel) 3. Read: [IDEO. (2008). What does design thinking feel like?](https://designthinking.ideo.com/blog/what-does-design-thinking-feel-like) |
| Class 7: Ideation (3/9) | Ideation Day 2  [co-creation session](https://www.designkit.org/methods/co-creation-session), [storyboarding](https://www.designkit.org/methods/storyboard), [role play](https://www.designkit.org/methods/role-play), [journey map](https://www.designkit.org/methods/journey-map), [ways to grow framework](https://www.designkit.org/methods/ways-to-grow-framework), [gut check](https://www.designkit.org/methods/gut-check), [determine what to prototype](https://www.designkit.org/methods/determine-what-to-prototype)[, build and run prototypes](https://www.designkit.org/methods/build-run-prototypes), [rapid prototyping](https://www.designkit.org/methods/rapid-prototyping), [get feedback](https://www.designkit.org/methods/get-feedback), [integrate feedback and iterate](https://www.designkit.org/methods/integrate-feedback-and-iterate), [create a logic model](https://www.designkit.org/methods/create-a-logic-model), [explore your theory of change](https://www.designkit.org/methods/explore-your-theory-of-change), [business model canvas](https://www.designkit.org/methods/business-model-canvas) | Required:   1. Read: Review methods for this week in *The Field Guide for Human-Centered Design* or the [Design Toolkit](https://www.ideo.com/post/design-kit) 2. Watch/Listen: [Prototyping Insights + The Prototyping Canvas with Carlye Lauff — DT101 E46](https://fluidhive.com/prototyping-insights-the-prototyping-canvas-with-carlye-lauff-dt101-e46/)   Recommended:   1. Read: Andrew Askins and Laura Bosco. (n.d.) [5 examples of no-code prototypes: Invision, spreadsheets, and more.](https://krit.com/blog/five-examples-of-no-code-prototypes) 2. Watch: IDEOU. [Why should you prototype?](https://ideou.wistia.com/medias/6bpjit9ka5) 3. Watch: IDEO. [Redesigning how Coca-Cola Works, Plays, and Profits.](https://www.ideo.com/case-study/redesigning-the-way-coca-cola-works-plays-and-profits) |
| Class 8: Ideation (3/16) | Guest Lecture and Design sprint #2  [Lost in Translation](https://transformativetoolkit.org/activity/lost-in-translation) activity | Required:   1. Read: TBD 2. Watch/Listen: [Teams, Sprints, Prototyping, and Better Meetings with Douglas Ferguson — DT101 E59](https://designthinking101.libsyn.com/teams-sprints-prototyping-and-better-meetings-with-douglas-ferguson-dt101-e59) |
| Class 9: Ideation (3/23) | Group Project planning time (2+ hours)  *Individual Assignment #2 due* | Required:   1. Read: [Ricard, S. (2020, January 08). Council Post: Five Strategies To Improve Communication With Team Members](https://www.forbes.com/sites/forbestechcouncil/2020/01/08/five-strategies-to-improve-communication-with-team-members/). 2. Watch/Listen: [Designing Your Team + Teams in Design Education + Coaching Design Teams with Mary Sherwin and David Sherwin — DT101 E49](https://fluidhive.com/designing-your-team-teams-in-design-education-coaching-design-teams-with-mary-sherwin-and-david-sherwin-dt101-e49/)   Recommended:   1. Listen: [Weaving Design Thinking into Teams, Leaders and Organizations with Holly O’Driscoll — DT101 E51](https://fluidhive.com/weaving-design-thinking-into-teams-leaders-and-organizations-with-holly-odriscoll-dt101-e51/) 2. Listen: [HBR Ideacast: How Personalities Affect Team Chemistry](https://hbr.org/podcast/2017/03/how-personalities-affect-team-chemistry) |
| Class 10: Ideation (3/30) | Group Project execution time with end users (3 hours) | No required readings. |
| Class 11: Implementation (4/6) | Implementation overview  [Live prototyping](https://www.designkit.org/methods/live-prototyping), [pilot](https://www.designkit.org/methods/pilot), [optimize and adapt for scale](https://www.designkit.org/methods/optimize-and-adapt-for-scale), [create a pitch](https://www.designkit.org/methods/create-a-pitch), [keep iterating](https://www.designkit.org/methods/keep-iterating), [keep getting feedback](https://www.designkit.org/methods/keep-getting-feedback)  [Gallery of Failure](https://transformativetoolkit.org/activity/gallery-of-failure) activity  *Lenses and Power Individual Reflection due on Sakai Forum* | Required:   1. Read: Review methods for this week in *The Field Guide for Human-Centered Design* or the [Design Toolkit](https://www.ideo.com/post/design-kit) 2. Watch/Listen: [Growth Mindset vs. Fixed Mindset](https://www.youtube.com/watch?v=M1CHPnZfFmU)   Recommended:   1. Watch: [Developing a Growth Mindset with Carol Dweck](https://www.youtube.com/watch?v=hiiEeMN7vbQ) 2. Read: [What Having a "Growth Mindset" Actually Means](https://hbr.org/2016/01/what-having-a-growth-mindset-actually-means) |
| Class 12: Implementation (4/13) | Group Project planning and execution time with end users (3 hours)  *Growth Mindset Individual Reflection due on Sakai Forum* | Required:   1. Read: Brett and Stephen B. Goldberg, J., Gallo, A., Maimon, A., & Ashkenas, R. (2017, November 29). [How to Handle a Disagreement on Your Team.](https://hbr.org/2017/07/how-to-handle-a-disagreement-on-your-team) 2. Watch/Listen: [Ray Dalio - How to embrace conflict](https://podbay.fm/podcast/1227971746/e/1574737200)   Recommended:   1. Read: Llopis, G. (2020, August 31). [4 Ways Leaders Effectively Manage Employee Conflict.](https://www.forbes.com/sites/glennllopis/2014/11/28/4-ways-leaders-effectively-manage-employee-conflict/) 2. Watch: Lindred Greer: [Managing Conflict in Teams](https://www.youtube.com/watch?v=YVWpipM-Rnw) |
| Class 13: Implementation (4/20) | [Roadmap for success](https://www.designkit.org/methods/roadmap-for-success), [capabilities quicksheet](https://www.designkit.org/methods/capabilities-quicksheet)  [build partnerships](https://www.designkit.org/methods/build-partnerships), [staff your project](https://www.designkit.org/methods/staff-your-project), [funding strategy](https://www.designkit.org/methods/funding-strategy), [sustainable revenue](https://www.designkit.org/methods/sustainable-revenue), [explore scalability](https://www.designkit.org/methods/explore-scalability), [define your indicators](https://www.designkit.org/methods/define-your-indicators), [monitor and evaluate](https://www.designkit.org/methods/monitor-and-evaluate)  *Group Ideation Report due*  *Conflict Individual Reflection due on Sakai Forum* | Required:   1. Read: Review methods for this week in *The Field Guide for Human-Centered Design* or the [Design Toolkit](https://www.ideo.com/post/design-kit) 2. Watch/Listen: [How I Built This. Away – Jen Rubio.](https://www.npr.org/2019/03/08/701651787/away-jen-rubio)   Recommended:   1. Read: IDEO. [Helping Investors Align Their Dollars With Their Values](https://www.ideo.com/post/helping-investors-align-their-dollars-with-their-values). |
| Class 14: Implementation (4/27) | Group Project planning and execution time with end users (3 hours)  [Pre-Mortem](https://transformativetoolkit.org/activity/pre-mortem) activity | Required:   1. Read: Boogaard, K. (2020, June 19). [How to Take Feedback Like a Pro.](https://www.themuse.com/advice/how-to-take-feedback-like-a-pro) 2. Read: Muse. [5 Steps to Giving Good Feedback.](https://www.themuse.com/advice/5-steps-to-giving-good-feedback) 3. Watch/Listen: [7 Tips for Accepting Feedback.](https://www.youtube.com/watch?v=Qo6IBY2WR6o)   Recommended:   1. Watch: [The power of vulnerability](https://www.youtube.com/watch?v=iCvmsMzlF7o) 2. Read: Eurich, T. (2018, August 06). [The Right Way to Respond to Negative Feedback.](https://hbr.org/2018/05/the-right-way-to-respond-to-negative-feedback) 3. Read: [Michigan State University. Requesting and Accepting Feedback.](https://hr.msu.edu/performanceexcellence/tools-staff-feedback.html) |
| Class 15: Implementation (5/4) | Group Project planning for final submission and presentation  *Feedback Individual Reflection due on Sakai Forum*  Final presentations | No required readings. |
| Final Exam Block: May 11^th^ from 7:00pm-10:00pm | *Group Implementation Report due by noon*  Final presentations | No required readings. |

**Course Assignments and Assessments**

This course will include the following graded assignments that contribute to your final grade in the course. For assignment descriptions and assignment grading rubrics, please see the Sakai course site.

| **Graded Assignments** | **Percentages of**  **Final Course Grade** |
| --- | --- |
| 1. Individual Reflections | 10% |
| 1. Individual Inspiration Assignment | 5% |
| 1. Group Design Sprint #1 | 10% |
| 1. Group Project Inspiration Report (part of final project) | 10% (5% group grade and 5% individual group contribution grade) |
| 1. Individual Ideation Assignment | 10% |
| 1. Group Design Sprint #2 | 10% |
| 1. Group Project Ideation Report (part of final project) | 15% (10% group grade and 5% individual group contribution grade) |
| 1. Participation | 10% |
| 1. Final Project (group) | 20% (15% group grade and 5% individual group contribution grade) |
| **TOTAL** | **100** |

**Syllabus Appendix**

**Course Grading Scale(s)**

Final course grades will be determined using the following [UNC-CH Graduate School grading scale](http://handbook.unc.edu/grading.html). The relative weight of each course component is shown in the Graded Assignments table.

|  | **Description** | **Numeric Value** |
| --- | --- | --- |
| **H** | High Pass: Clear excellence | 93-100 |
| **P** | Pass: Entirely satisfactory graduate work | 80-92 |
| **L** | Low Pass: Inadequate graduate work | 70-79 |
| **F** | Fail | 0-69 |

**Instructor Expectations:**

**Email**

Instructors will typically respond to email within 48 hours if sent Monday through Friday. Instructors may respond to weekend emails, but it is not required of them. If you receive an out of office reply when emailing, it may take longer to receive a reply. Instructors will provide advance notice, if possible, when they will be out of the office.

**Feedback**

All graded assignments will receive written feedback that coincides with the assessment rubric. Feedback is meant to be constructive and help the student continue to build upon their skills. The types of feedback you may receive are descriptive feedback, evaluative feedback, and motivational feedback. Feedback is a tool that you as a learner can use to understand the areas that you are succeeding in and what you can do to improve in other areas.

**Grading**

Instructors aim to grade and return assignments within two weeks of the due date. Early submissions will not be graded before the final due date.

**Honor Code**

Instructors may report suspected violations of the Honor Code, including inappropriate collaborative work or problematic use of secondary materials, to the Honor Court. Honor Court sanctions vary but may include receiving a zero for the assignment, failing the course and/or suspension from the university. See Additional Resources and Policies for additional information.

**Inclusive Excellence**

In this class, we practice the UNC-CH’s commitment to inclusion, diversity, and equity in the following ways. See Additional Resources and Policies for additional information.

- Develop classroom participation approaches that acknowledge the diversity of ways of contributing to the classroom and foster participation and engagement of *all* students.
- Structure assessment approaches that acknowledge different methods for acquiring knowledge and demonstrating proficiency.
- Encourage and solicit feedback from students to continually improve inclusive practices.

**Syllabus Changes**

The instructor reserves the right to make changes to the syllabus, including topics, readings, assignments, and due dates. Any changes will be announced as early as possible. For session-by-session course schedule details, please see the Sakai course site.

**Student Expectations:**

**Appropriate Use of Course Resources**

The materials used in this class, including, but not limited to, syllabus, class activities, and assignments are copyright protected works. Any unauthorized copying of the class materials is a violation of federal law and may result in disciplinary actions being taken against the student. Additionally, the sharing of class materials without the specific, express approval of the instructor may be a violation of the University's Student Honor Code and an act of academic dishonesty, which could result in further disciplinary action. This includes, among other things, uploading class materials to websites for the purpose of sharing those materials with other current or future students.

**Assignments**

Submit all assignments through the Assignments or Dropbox features on Sakai in accordance with assignment instructions. Emailing assignments is not acceptable unless prior arrangements have been made. If you are having issues submitting assignments, try a different web browser first. If switching browsers does not work, email, or call the instructor for guidance.

All submitted assignments should use the following file naming convention:

LastnameFirstinitial_Assignmentname.docx

Example: ChenE_Individual_Inspiration_Assignment.docx

For group project assignments, only one group member needs to submit the assignment using the Assignments feature on Sakai. Please use your group project team name in the file naming convention:

Teamname_Assignmentname.docx

Example: TeamGophers_Inspiration_report.docx

For design sprints, only one group member needs to submit the assignment using the Assignments feature on Sakai. Please use your group’s assigned number in the file naming convention:

Group#_Assignmentname.docx

Example: Group3_Design_Sprint_#1.docx

**Attendance**

Your active participation and engagement are an integral part of your learning experience in this course. We know that attending class sessions synchronously (i.e., live) will not always be possible. If you are not able to join class synchronously, please email Dr. Chen, Ms. Jagannathan, and Ms. Skywark as soon as possible so that they can provide you with information for how to access and complete class activities that were done synchronously. We want to ensure that all students have access to the content and learning experiences provided.

Please also see the University’s [Absences Related to Covid-19 policy](https://attendance.unc.edu/absences-related-to-covid-19/).

**Communication**

You are expected to follow common courtesy in all communication to include email, class discussions, and office hour appointments. All electronic communications sent should follow proper English grammar rules to include complete sentences. This is a professional course, and you are expected to communicate as a professional.
